# The space of enzyme regulation in HeLa cells can be inferred from its intracellular metabolome

**DOI:** 10.1038/srep28415

**Published:** 2016-06-23

**Authors:** Christian Diener, Felipe Muñoz-Gonzalez, Sergio Encarnación, Osbaldo Resendis-Antonio

**Affiliations:** 1 Instituto Nacional de Medicina Genómica (INMEGEN), Mexico City 14610, Mexico; 2 Centro de Ciencias Genómicas, Universidad Nacional Autónoma de México, Cuernavaca 62210, México; 3 Coordinación de la Investigación Científica - Red de Apoyo a la Investigación UNAM, Mexico

## Abstract

During the transition from a healthy state to a cancerous one, cells alter their metabolism to increase proliferation. The underlying metabolic alterations may be caused by a variety of different regulatory events on the transcriptional or post-transcriptional level whose identification contributes to the rational design of therapeutic targets. We present a mechanistic strategy capable of inferring enzymatic regulation from intracellular metabolome measurements that is independent of the actual mechanism of regulation. Here, enzyme activities are expressed by the space of all feasible kinetic constants (k-cone) such that the alteration between two phenotypes is given by their corresponding kinetic spaces. Deriving an expression for the transformation of the healthy to the cancer k-cone we identified putative regulated enzymes between the HeLa and HaCaT cell lines. We show that only a few enzymatic activities change between those two cell lines and that this regulation does not depend on gene transcription but is instead post-transcriptional. Here, we identify phosphofructokinase as the major driver of proliferation in HeLa cells and suggest an optional regulatory program, associated with oxidative stress, that affects the activity of the pentose phosphate pathway.

During the development of cancer, cells undergo major metabolic changes to increase their capacity for proliferation. In many cases, that transition is characterized by an increased usage of fermentation, which is independent of the presence of oxygen and caused by a higher flux through glycolysis and diminished activity of the TCA cycle^1^. The resulting decreases in respiration and secretion of lactate have been known since the 1920s and were named after Otto Warburg. Although the Warburg effect has been well characterized in the last century, questions remain as to which regulatory changes are necessary to cause it^2^. The recently increased availability of genome and proteome technologies has provided great advantages in the analysis of genome-level regulation during the formation of cancer^3^,^4^,^5^. Thus, it has become apparent that regulatory events in cancer development are heterogeneous and that the regulatory events causing the Warburg effect may be distinct between different cancer types or even patients with the same cancer^6^,^7^. Additionally, data on the transcription level may only detect a subset of regulatory events, such as changes in gene expression and mutations, but fail to find post-translational regulation events such as protein modifications, phosphorylation or allosteric regulation that might have a large impact on cancer development^8^,^9^,^10^,^11^.

Therefore, it comes as no surprise that there has been an ongoing effort to combine data from the genome with the metabolome, the concentrations of all of the cells’ metabolites^12^,^13^. Metabolome data are inherently more complicated to obtain than genome data due to the necessity of different protocols for different metabolites. However, the metabolome is also closer to the cellular phenotype, as it forms the basis for growth and cellular health and has proven to deliver reliable markers for the detection of cancer^12^,^14^. This case holds particularly for the intracellular metabolome which gives a detailed snapshot of a cells’ metabolic state and is often more informative than extracellular measurements from biofluids^15^.

While there exists a wide selection of methods to analyze genomic data and infer regulation events in cancer up to the enzyme level, metabolome data are often analyzed solely on the abundance level with only limited ability to extend this information to the entire biological system or even connect metabolome data to the inferences made from genome data. However, there exist some notable exceptions where methods from Systems Biology have been applied successfully^16^,^17^. Here, a common strategy is to employ fluxome analysis, based mostly on Flux Balance Analysis (FBA) or control theory^18^,^19^,^20^,^21^,^22^. FBA has been shown to be a valuable tool in cancer research, albeit with certain limitations^23^. In particular, it is difficult to connect metabolome data to FBA because FBA does not treat kinetics explicitly and therefore has no direct quantifiable concept of how metabolite concentrations influence cellular fluxes. An alternative formulation termed “k-cone” remedies this situation by acting on the space of possible kinetic parameters rather than fluxes^24^,^25^,^26^. In contrast to FBA, it makes assumptions about specific kinetics and may give better insight into the systems dynamics than FBA. In the analysis of regulation events, k-cone analysis is particularly useful because it expresses the systems properties in terms of individual enzyme activities, which can be connected to other omics data such as genome or proteome data that give estimates of enzyme concentrations.

In this manuscript, we present the first metabolome profiles for the cancerous HeLa and non-cancerous HaCaT cell lines. We use the k-cone formalism to obtain a differential analysis of enzyme activities between the two cell lines, as that formulation can use metabolome data to provide a mathematical transformation between the normal and disease state. Our analysis identifies alterations in the enzyme activities of HeLa cells, many of which are consistent with previously identified changes. Furthermore, we also show that there exists a set of optional enzyme regulations that may help HeLa cells to alleviate oxidative stress without compromising proliferation. Taken together, we propose that differential k-cone analysis, which may integrate genome-scale metabolic reconstructions and metabolome data, is a suitable conceptual scheme to identify and suggest the regulatory mechanisms required to establish the phenotype in cancer cell lines.

## Results

### Obtaining the k-cone and global stability from metabolome data

K-cone analysis is closely related to the steady state, the state of homeostasis for a metabolic system. During the steady state, metabolite concentrations are constant which gives rise to the steady state equation


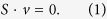


Here, S denotes the stoichiometric matrix and v the vector of steady state fluxes. The space of all v that fulfill that equation is commonly known as the flux cone. The k-cone is based on the same equation but further assumes a distinct structure of the fluxes, where each flux can be decomposed into a kinetic constant and a metabolic term given by the metabolite concentrations x as v_i_ = k_i_m_i_(x). This case holds for mass-action kinetics with stoichiometries s_ji_


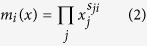


and gives rise to the k-cone equation


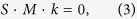


where M denotes a diagonal matrix containing the mass-action terms m_i_(x) on its diagonal^24^,^26^. This equation now defines a space for all feasible kinetic constants in the steady state, the k-cone. Dividing all reversible biochemical reactions into their irreversible individual forward and backward reactions further yields a k-cone that is strictly positive. It is important to note that the k-cone does not identify the exact kinetic constants for the system but rather the space in which those constants must reside. Furthermore, the k-cone can be constrained by known *in vivo* equilibrium constants (see Supplementary Text)^24^. Because we were particularly interested in a differential analysis of enzyme activities, we tried to find expressions relating the k-cone of a normal cellular state to a disease state. As we show in detail in the Supplementary Text, given the k-cones for the normal state K_n_ and the disease state K_d_, the corresponding mass action term matrices M_n_ and M_d_ define a diagonal transformation matrix T = M_n_M_d_^−1^ such that K_d_ = TK_n_. Thus, the difference between the normal and the disease state of the entire space of steady state kinetic parameters is completely defined by the mass-action terms which can be obtained from metabolome data. The diagonal matrix T explains all possible changes in enzyme activity between the normal and disease state in a quantitative manner and quantifies the prevalence with which an enzyme is regulated when the fluxes are unknown. Specifically, if feasible kinetic constants were randomly sampled from the normal and disease k-cones for the same cell, the expectation of their fold changes would be given by the diagonal of T, which is why we will denote the entries in T as the expected differential activities (EDAs).

However, one particular normal or disease state only occupies one distinct point, k_n_ or k_d_, in its respective k-cone, K_n_ or K_d_. In the case where the fluxes are known along with the metabolome one can use the mass-action kinetics, v_i_ = k_i_m_i_(x), to pinpoint the kinetic constants directly through dividing the fluxes by the corresponding mass-action terms m_i_(x). Based on this argumentation, one can derive a relation that incorporates flux data and where k_d_ = TWk_n_ (Supplementary Text). Here, W is a diagonal weight matrix containing the steady state flux ratios v_d_/v_n_. Thus, the transformation of the entire k-cone is given by metabolome data alone, whereas the exact position in the respective k-cones is defined by fluxome data. The fluxome of human cell lines is usually unknown, but there exist several strategies for using prior information to estimate a feasible flux distribution of cancer cells. Here, the most common strategy is to use flux balance analysis (FBA), possibly incorporating other genome-scale data, such as gene expression and growth rate measurements^27^.

**Figure 1:**
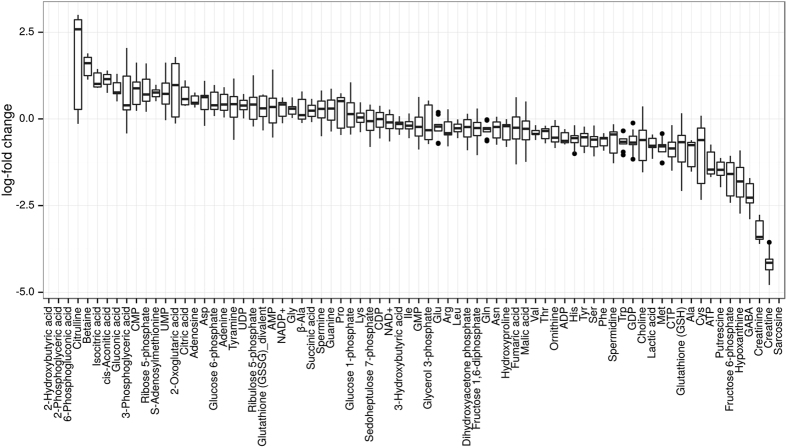
Differential metabolome profile of HeLa versus HaCaT cells. The log_2_-fold changes between HeLa and HaCaT cells (3 biological replicates each). The boxplots denote the distribution of log-fold changes for all combinations of HeLa and HaCaT samples. Missing boxes denote metabolites that could not be detected. Some replicates also showed missing values and the full data set can be found in Supplementary Table S1.

Metabolome measurements were obtained for the HaCaT keratinocyte and HeLa cell lines, which are both selected for their ability to proliferate independently and have very similar doubling times^28^,^29^,^30^. Here, HaCaT cells were used as an immortalized control, meaning the cell line employs unlimited proliferation but is not cancerous, whereas HeLa is a cervical cancer cell line. We quantified a large fraction of the metabolites participating in the central carbon metabolism for both cell lines (three biological replications for each cell line, data in Table S1, see Materials and Methods). The obtained log-fold changes in metabolite concentrations are shown in Fig. 1 and were consistent with previously published works^15^,^31^,^32^ on different cancers, particularly in showing a strong deregulation of metabolites and intermediates of the glycine and proline metabolisms between HeLa and HaCaT^33^.

The measured metabolite concentrations were mapped to a model of the central carbon metabolism, extending a previously published model to yield one with 100 irreversible reactions assuming mass-action kinetics^34^. The model contained all major pathways of central carbon metabolism, such as glycolysis/gluconeogenesis, the TCA cycle and the pentose phosphate pathway, as well as simplified versions of cellular respiration and oxidative stress (Table S2). Of the measured metabolites, 28 could be mapped to the 43 metabolites in the model and the remaining unmapped model metabolites were imputed by previous measurements and assumed to be the same in HaCaT and HeLa (see Supplementary Tables S1 and S2 as well as Materials and Methods). This yielded the 100 mass-action terms of the model, where 71 mass-action terms were based on at least one measured (non-imputed) metabolite and 43 mass-action terms contained at least one imputed metabolite.

**Figure 2:**
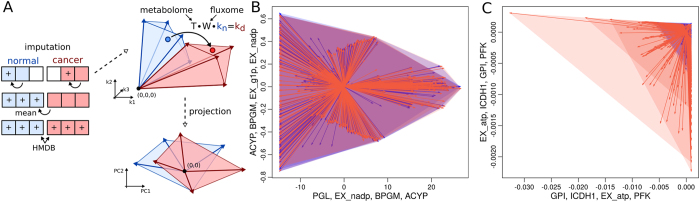
The k-cone space of HaCaT and HeLa cells. (**A**) Missing data are imputed within or across conditions using the measured concentrations. The “+” sign denotes newly imputed values. Only metabolites completely lacking from measurements are obtained from the HMDB database. Enzyme activities in a disease state are given by a distinct transformation of enzyme activities from a healthy reference, composed of a transformation matrix T, which transforms the entire k-cone space and is given by metabolome data alone, and a weight matrix W, which maps the distinct points of enzyme activities in the k-cone spaces and requires fluxome data. For visualization purposes, the k-cone spaces were reduced to the two dimensions explaining the highest variance. (**B**) Projection of k-cone spaces for HaCaT metabolome measurements (blue) and HeLa (red). The axes correspond to the two largest eigenvectors of the entire space and the annotations denote reactions with the highest absolute loadings in those eigenvectors. Arrows denote the clusters of basis vectors of the respective k-cones and shaded areas denote the space within the k-cone; thus, all points falling in this area denote feasible enzyme activities (3 measurements for each cell line). The relative error of the dimension reduction is 6.7%. The mean distance within arrow clusters is 0.13. (**C**) K-cone spaces for only those reactions whose mass-action terms changed at least by a factor of 2. The relative error of the projection was 6.9%, and the mean distance within arrow clusters was 2.8e-5.

Using this model, we derived the specific k-cones and transition matrix T from the metabolite measurements, yielding a k-cone containing over 80,000 basis vectors. Due to the high dimensionality of the k-cone visualization requires a mapping to a lower dimensional space. We employed two different strategies for this purpose. In the first attempt, shown in Fig. 2B, we mapped the reduced k-cone onto two dimensions by principal component analysis (PCA) and proceeded by clustering the reduced k-cone vectors in order to eliminate identical vectors in the reduction (also see Materials and Methods for details on the projection). As an alternative we also employed a strategy based on reducing the original k-cone space. Here, we used additional constraints taken from approximations of *in vivo* equilibrium constants (K_eq_) obtained from the equilibrator database^35^ (http://equilibrator.weizmann.ac.il/) which reduced the k-cone to only 40 basis vectors which were then mapped onto two dimensions by PCA (see Fig. S1, Table S2 and Supplementary Text). We observed that on a global scale, the space of enzyme activities, as given by the kinetic parameters, was almost identical between the HaCaT and HeLa cell lines, indicating that the differences were limited to reactions with relatively low enzyme activities (compare Fig. 2B and Fig. S1A). To see whether there exists a subspace where the k-cones differ, we also visualized the k-cone for only those reactions whose mass-action terms changed by at least 2-fold, thus indicating large influences in the matrix T (Fig. 2C and S1B). Here, we observed a stronger difference between the k-cone spaces of the normal and control group, mostly achieved by slight rotation and strong scaling in the basis vectors. Those results could be observed for the complete k-cones as well as for the ones constrained by equilibrium constants.

Given a k-cone basis, it is also possible to evaluate the stability of the entire basis. Here, stability denotes the ability of the system to return to its steady state upon slight perturbation. For a detailed explanation refer to Supplementary Text. We calculated the stability for all basis vectors in each of the non-reduced k-cones. Because every possible steady state solution of kinetic constants must be a linear combination of the k-cone basis vectors, the global stability of the system must be a combination of the observed stabilities. The majority of all k-cone basis vectors were stable, which is to be expected of biologically relevant steady states (Fig. S1C). However, there was also a large group of unstable basis vectors, albeit with very small positive eigenvectors. One should note that due to the smaller absolute values of eigenvalues in the unstable states, the stable state will predominate, meaning that mixed states composed of stable and unstable basis vectors will most likely be stable (see Supplementary Text). The proportion of stable basis vectors in each k-cone remained the same between HaCaT and HeLa cells, suggesting that cancer in HeLa cells is essentially a stable state and as difficult to perturb as the non-cancerous HaCaT cells.

### Inference of enzymatic regulation in HeLa

As shown in the previous section, the transition given by T gives an approximation of enzyme activity fold-changes between the HeLa and HaCaT cell lines and does not require explicit calculation of the k-cone. However, because the mass-action terms are based on the products of noisy data, special care must be taken to avoid an influence of the reaction order (number of multiplicands in the mass-action terms) on the fold-changes and their statistics. Thus, we performed all analyses in log-space considering log_2_T rather than T. The problem of identifying differential enzyme activity is similar to the problem of identifying differential gene expression. Thus, analysis methods for microarray data can be applied to the mass-action terms if they follow the required log-normal distribution. Because we used mass-action kinetics, the logarithmic transformation of the mass-action terms is a weighted sum of the log-transformed metabolite concentrations and is approximately normally distributed as long as the log-transformed metabolite concentrations are as well. The validity of this assumption was verified by quantile-quantile plots as well as the empirical distribution function of the log-transformed metabolite concentrations (shown in Fig. S2). This assumption allowed us to employ methods from microarray analysis, as used by the limma package^36^. The significance of the observed log-fold changes in enzyme activity between HaCaT and HeLa cells was obtained by Welch t-tests using an empirical Bayes estimator for the stable quantification of sample variances^37^.

There is the possibility that only a subset of the reactions are actually required to maximize proliferation, leaving a large window of variation for fluxes that do not directly influence the growth rate. Due to the identical model, and thus identical flux cone, for both conditions, those differences would not be detectable with standard methods to calculate metabolic fluxes such as sampling from the flux cone or flux balance analysis that would both result in the same fluxes for both conditions in average. Because the accuracy of our approximation of differential enzyme activity is compromised by large changes in the fluxes between the cell lines, we performed flux variability analysis to identify the maximum log-fold change that could be caused by variation in the fluxes. Here, we first added a biomass reaction to the model and maximized its flux. This step was followed by flux variability analysis to obtain upper bounds for the absolute log-fold change for each reaction flux^38^. The individual log-fold changes were then filtered by those upper bounds to leave only those log-fold changes that could not be counteracted by flux variation (see Materials and Methods). This process could be used to identify differential enzyme activities that were necessary for optimal proliferation, as they could not be explained by flux variation under optimal growth. This analysis can be interpreted as identifying dimensions in the k-cones that do not overlap between normal and disease conditions.

**Figure 3:**
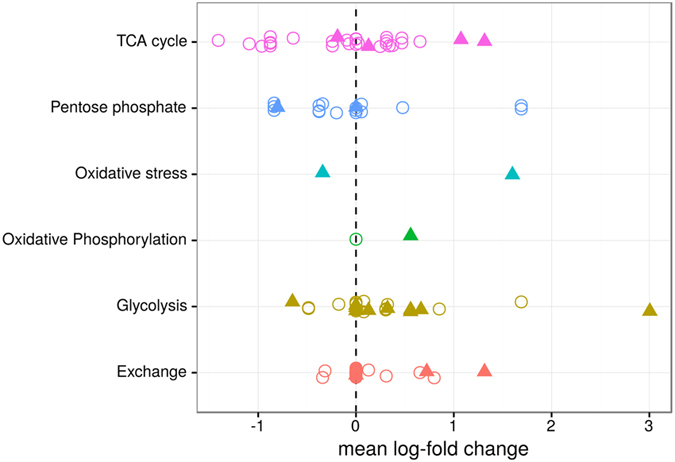
Global changes in enzyme activity between HeLa and HaCaT cells. Changes in enzyme activities are shown as mean log_2_-fold changes between HeLa and HaCaT cells. Each point denotes a single irreversible reaction, and reactions are grouped by metabolic pathway. Circles denotes the expected differential enzyme activities predicted by the transformation matrix T, and triangles denote regulations that are necessary for proliferation.

**Figure 4:**
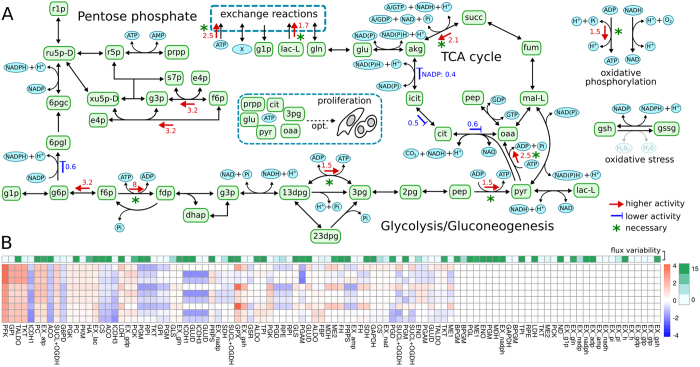
Reaction-level differences of enzymatic activity. (**A**) Reactions with significantly (corrected p < 0.05) altered enzyme activities between HeLa and HaCaT cell lines are indicated by colored arrows in the direction of the alteration together with their mean fold changes. Regulations that are necessary for proliferation (worst case corrected p < 0.05) are marked with green asterisks. (**B**) Log-fold changes between all combinations of HeLa and HaCaT samples (3 biological replicates each). Colors from blue to red denote log-fold changes and columns are sorted by increasing p-values from left to right. The upper bounds for the fluxes absolute log-fold changes as estimated from flux variability analysis are shown in green.

The mean log-fold changes for the EDAs in each reaction are shown in Fig. 3. Reactions with a significant p-value (FDR corrected p < 0.05) in their EDAs are indicated in Fig. 4A, and their individual log-fold changes along with their flux variation are shown in Fig. 4B. Mean log fold changes along with their credible intervals are reported in Supplementary Table S3.

Notably, many of the enzymes with significant EDAs are already known to be altered in cancer. Those enzymes include phosphofructokinase, phosphoglycerate mutase, pyruvate kinase, 6-phosphogluconate dehydrogenase, pyruvate dehydrogenase and aconitase^39^,^40^,^41^,^42^,^43^,^44^,^45^. Previously unknown regulation events include a retention of glucose-6-phosphate, an increased production rate of PRPP (5-Phospho-alpha-D-ribose 1-diphosphate) and a strongly accelerated production of glyceraldehyde 3-phosphate. Additionally, the EDAs predict a strong dysregulation of TCA cycle enzyme activities as well as increased ATP usage and lactate export in HeLa cells, all consistent with the Warburg effect^14^. The set of necessary regulations for proliferation consisted of the up-regulation of four glycolytic enzymes, the up-regulation of ATP synthesis and the increased export of lactate. Thus, the Warburg effect in HeLa cells seems to be a consequence of maintaining a high proliferation rate. In general, the strongest regulation was observed for phosphofructokinase, which showed an 8-fold increase in enzyme activity in HeLa compared to the HaCaT cell line. Because phosphofructokinase is allosterically regulated by ATP, citrate and pH, this regulation is consistent with the observed lower concentrations of ATP, citrate and lactate as shown in Fig. 1^ ^46.

### Relation to gene expression and coregulation of heterogeneous enzyme activities

**Figure 5:**
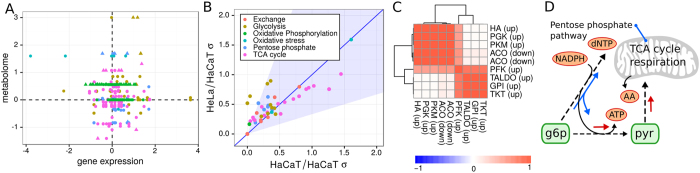
Heterogeneity and co-regulation in HeLa cells. (**A**) Comparison of the log-fold changes in enzyme activity by either gene expression analysis of 58 microarray samples (20 HaCaT/keratinocyte and 38 HeLa) or the EDAs obtained from metabolome data. Colors denote pathways and are the same as in Fig. 2 and panel B of this figure. Triangles denote enzymes that are significantly regulated in their EDAs and gene expression. (**B**) Standard deviations of HeLa/HaCaT log-fold changes as obtained after optimizations. Each point denotes a reaction, and the colors are the same as used in Fig. 2. The blue-shaded area denotes a change in standard deviation from HaCaT to HeLa by a factor of 3. The blue line denotes a 1:1 relationship between standard deviations. (**C**) Correlation matrix for log-fold changes in differential heterogeneous reactions in HeLa cells, as obtained from the EDAs. Names denote enzymes and the type of regulation relative to HaCaT cells is denoted in brackets. (**D**) Illustration of the proposed major metabolic changes in HeLa cells. Proliferation in HeLa cells requires higher enzyme activities in glycolysis (mostly due to phosphofructokinase) and increased fidelity in the entry to the TCA cycle (red arrows). Additionally, higher activity in the TCA cycle and respiration lead to up-regulation of the pentose phosphate pathway which is drained into NADPH, nucleotide precursors and glycolytic intermediates (blue arrows).

Given our predictions of differential enzyme activity based on metabolome data (EDAs), we also investigated how well these data would correlate with differential gene expression in HaCaT and HeLa cells and, thus, whether the observed changes in enzyme activity are due to changes in gene expression. For this purpose, we assembled a data set consisting of 58 microarray samples (20 HaCaT and keratinocytes and 38 HeLa) on a single platform (HGU133Plus 2.0) obtained from the GEO database^47^. Here, the keratinocyte samples were added because only very few HaCaT samples were available in the GEO database. Their validity was checked by PCA and clustering over the expression values, which consistently grouped the keratinocyte samples together with the HaCaT samples (Fig. S2 and Supplementary Text). All samples in the list were curated manually to ensure that they described untreated conditions, and they can be found in Supplementary Table S4. We found that log-fold changes obtained from EDAs and gene expression did not correlate on a global level (see Fig. 5A, Pearson product-moment correlation <0.01, p > 0.83). However, some of the enzymes with the largest changes in activity also showed significant changes in gene expression (compare Table S5), particularly phosphofructokinase, glucose-6-phosphate isomerase and phosphoglucokinase. As shown in Fig. 5A, enzymes participating in the pentose phosphate pathway and TCA cycle with a significant change in enzyme activity, as predicted by metabolome data, often showed significant changes in gene expression, but in many cases in the opposite direction (meaning they were up-regulated in their EDA but down-regulated in gene expression and *vice versa*; also see Supplementary Text). In total, genomic regulation is mostly active in glycolysis. However, gene expression in general is not a good predictor of differential enzyme activity, suggesting that the primary regulation of metabolism in HeLa cells occurs on the post-transcriptional level.

Genomic analysis of human cancers has already shown that metabolic regulation in cancer can be highly heterogeneous and varies greatly among different cancers and even within patients with the same cancer^7^. Here, we aimed to analyze the heterogeneity of regulation on a metabolic level. Log-fold changes of enzyme activity within HaCaT samples and between HeLa and HaCaT samples both showed strong variations (compare Figs 3 and 4B). To analyze this phenomenon in more detail, we calculated standard deviations for all obtained log-fold changes of the EDAs for the control log-fold changes (within HaCaT) and differential log-fold changes (between HeLa and HaCaT). Here, standard deviations were mostly conserved on a reaction level between the HeLa and HaCaT cell lines, and most standard deviations for enzyme activities from the HeLa samples remained in the range of 3-fold (0.33 to 3) standard deviations within the HaCaT samples (see Fig. 5B). The largest variations within HaCaT and between HeLa and HaCaT could be observed for reactions alleviating oxidative stress and the reactions of the TCA cycle, indicating that both HaCaT and HeLa cells show variations in the regulation of enzymes involved in oxidative stress and the TCA cycle. Although gene expression was not a good predictor of enzyme activity changes, the heterogeneity seemed to be consistent with previously reported measurements of gene expression which also identified the TCA cycle and oxidative stress genes as the most heterogeneous ones in both normal and cancer cells^7^.

To identify reactions with specifically increased heterogeneity in cancer, we selected reactions whose variation increased by at least 3-fold in HeLa cells compared to HaCaT cells. For the EDAs, this selection produced a set of 11 reactions involved in the pentose phosphate pathway, glycolysis and respiration as well as increased ATP usage (see Fig. 5C). Log-fold changes of those reactions were highly correlated and formed two blocks, one connecting a high ATP usage, respiration and the late phase of glycolysis and another connecting reactions of the pentose phosphate pathway. Both of these blocks were connected by phosphofructokinase (PFK), showing a strong influence of PFK in the balancing of respiration with the pentose phosphate pathway.

Because in our results phosphofructokinase up-regulation is the most prominent change necessary for proliferation, we suggest the mechanism illustrated in Fig. 5D, where HeLa cells divert most of their metabolism towards glycolysis and use the pentose phosphate pathway only when a higher respiration requires it.

## Discussion

As we have shown, differential k-cone analysis is capable of suggesting regulated enzymes in the transition to cancer for the central carbon metabolism of HeLa cells. We feel that this method is particularly well-suited to examining the regulation of non-essential enzymes or enzymes that are not visibly affected on the genome level, as it detects regulation on the metabolome level, explicitly including post-transcriptional regulation events. Analysis based on the k-cone, as performed here, combines well with existing methods such as FBA or control theory by integrating data from the metabolome and thus giving a more appropriate description of the phenotype^48^. In particular, the quantities used in the k-cone method are very similar to FBA and create the possibility of incorporating prior knowledge in the form of the matrix W containing the flux ratios.

However, it is also important to note the limitations of our results. At least in the analysis we performed here, the method assumed the kinetics to be governed by the mass-action law, which is, at best, an approximation of the most likely more complex underlying kinetics. Another limitation is lacking metabolite measurements for metabolites included in the used model. The analyses, as we performed here, aimed to be conservative, meaning that we treated cases with missing data as non-differential.

Our results suggest that the activities of many enzymes in the central carbon metabolism of HeLa cells are similar to the activities found in the non-cancerous HaCaT cells. However, there is a small set of enzymes that can alter their activities in the two cell lines, and those changes do not seem to affect the stability of the system. Those regulation events can be further subdivided into a small set of necessary regulations required for proliferation and a slightly larger set of enzymes that can be regulated on a on-demand basis. Here, the up-regulation of phosphofructokinase (PFK) seems to be the major driver for maintaining proliferation, which explains the requirement of the Warburg effect, as a high concentration of lactate, citrate or unused ATP can allosterically inhibit phosphofructokinase^46^,^49^. As such, it might be beneficial for HeLa cells to limit the production of ATP in the TCA cycle to maintain a more active PFK. One of the optional regulation events in HeLa cells is a strong regulation of the entry and exits of the pentose phosphate pathway. This regulation caters to the needs of the cancer by draining the pentose phosphate pathway into ribonucleotide and glycolytic precursors while simultaneously producing NADPH, which is required to alleviate oxidative stress. Interestingly, our results propose an up-regulation of almost all enzymes in the central carbon metabolism using fructose 6-phosphate as a substrate, which might be associated with its necessity for glycolysis and the production of nucleotide precursors (compare Fig. 4). In our analysis, mitochondrial and oxidative stress enzyme activity show high variation in individual HeLa cell cultures and are correlated with pentose phosphate pathway enzyme activity (Fig. 5). Additionally, some of the genes associated with oxidative stress are among the most down-regulated ones (compare Fig. 5A and Table S5). This finding suggests a low tolerance of HeLa cells to oxidative stress in the default state, as most of glucose-6-phosphate is diverted into glycolysis by a more active PFK. However, this low tolerance can be counteracted by a high fidelity in deviating flux into the pentose phosphate pathway. There is some evidence that fidelity to oxidative stress is indeed due to the precedence of faster metabolic regulations^50^. However, at this stage, it is impossible to say whether this is an observation specific to the comparison of HeLa to HaCaT cells or a general mechanisms in various human cancers. There is some slight evidence of abnormal transaldolase activity in cancer, but not to the extent that we observed here^51^,^52^. Finally, experiments in yeast and *B. subtilis* suggest that large parts of the central carbon metabolism are regulated on a post-transcriptional level rather than on a transcriptional level, which seems to be the case particularly for the pentose phosphate pathway and late glycolysis, which we also find strongly regulated here^22^,^53^,^54^,^55^. Further investigation of this putative phenomenon could be of medical interest. Many of the more severe treatment options such as chemotherapy rely upon increasing oxidative stress in cancer cells. Thus, optional signatures of enzyme activity indicating a strong ability to combat oxidative stress via the pentose phosphate pathway might have consequences for the treatment options of those particular cancers.

We also observed that the metabolic differences between cancer and healthy cells are caused by the alteration of only a few enzymes. This result stands in contrast to mRNA measurements, which often suggest that cancer alters the expression of the majority of enzymes in the central carbon metabolism^7^. Thus, it seems that not all genomic aberrations are capable of affecting the dynamics of the underlying metabolic network sufficiently, and this result further outlines the necessity of methods that can map regulatory events in cancer to effects on metabolite abundances, which are closely connected to the resulting phenotype. Consequently, it would be worthwhile to combine the presented methods with existing omics data. For instance, one could study whether certain signatures in mRNA expression or protein abundance changes are associated with a particular change in enzyme activity. We feel that such a combination of methodologies could yield insights into the metabolic state of cancer cells and help understand their ruling principles, elucidate the heterogeneous causes of cancer and potentially identify new targets to halt or delay cancer progression.

## Materials and Methods

### Metabolome measurements

Measurement of the ionic metabolites was performed using the CE-MS system. The HeLa cell line was provided by the oncology laboratory of the Centro Medico Siglo XXI, which belongs to the Instituto Mexicano del Seguro Social. The HaCaT cell line was donated by the Centro de Investigación Sobre Enfermedades Infecciosas, which belongs to the Instituto Nacional de Salud Pública. Cell lines were cultured in RPMI-advanced as previously described by our group^56^. For each of the two cell lines, we obtained three biological replicates. First, 5 · 10^6^ cells were harvested at 70% confluence with 2 ml MeOH including the internal standards. Then, 1.6 ml of cell suspension was transferred to microcentrifuge tubes containing 1.6 ml of CHCl_3_ plus 640 μl of milliQ water, vortexed and then centrifuged at 2300 g and 4 °C for 5 minutes. 1.5 ml of the transferred aqueous layer was filtered through a Millipore 5-kDa cutoff and evaporated to dryness using a centrifugal evaporator. The measurement and quantification of extracted metabolites were performed by a commercial provider using a capillary electrophoresis (CE) connected to an ESI-TOF-MS with an electrophoresis buffer (Solution ID H3302-1021, Human Metabolome Technologies Inc., Tsuruoka, Japan).

Abundances were transformed into concentrations by dividing by the total volume of the 10^6^ cells, assuming an individual cell volume of 1.54 fl^57^. Hydrogen was not considered in the model due to lack of concentration (or intracellular pH) measurements.

Missing metabolite concentrations required in the model were imputed in multiple steps. First, missing data were imputed from measurements within the same cell line, followed by imputation across the two cell lines. Concentrations for metabolites that could not be detected in either of the two cell lines were obtained from the Human Metabolome database, primarily using cytosolic concentration measurements and falling back to blood measurements if cytosolic measurements were not available^58^. The actual values of those imputed concentrations were only of importance for the stability analysis described below. During differential analysis, metabolites with missing measurements for either cell line were assigned fold-changes of one due to the nature of the imputation procedure. As such, we made the implicit assumption that missing metabolite concentrations did not change across the two cell lines.

### Data availability and reproducibility

The raw data used for the analysis are provided in Supplementary Tables S1, S2 and S4. The methods of data handling, optimization and analysis were implemented in the dycone R package available at https://github.com/cdiener/dycone together with installation instructions (doi: 10.5281/zenodo.49987). A detailed protocol describing the steps taken to generate all figures and results in this paper is given in the Supplementary Text. To adhere to Open Science standards, the protocol is also available as R Markdown document together with the raw data files at https://github.com/cdiener/kcone-paper and can be used to reproduce all analyses in this manuscript.

### Model specification and k-cone calculations

The underlying kinetic model was obtained by extending a previously published model that had been validated by experimental data. The model was updated by annotating all reactions with their respective IDs from KEGG and adjusting the hydrogen balances to coincide with the ones reported in KEGG. We added additional reactions summarizing core mechanisms such as the neutralization of peroxide, import of glutamine, as well as the production of ATP and reduction of NADH in the mitochondria. Appropriate exchange reactions were added to molecules that could either be produced or consumed by metabolic processes not included in the model or obtained from the extracellular environment. The complete model specification can be found in Table S1, which is also the exact file read to generate the presented results.

A complete mathematical derivation of the formalism can be found in Supplementary Text. The k-cones for various metabolite measurements are obtained from the flux cone V. Because the flux cone equations define the H-representation of a polyhedral cone, obtaining the basis for the flux cone is equivalent to the vertex enumeration problem, which was solved using the method of Fukuda *et al*.^59^ using the Rcdd package (https://cran.r-project.org/web/packages/rcdd) on a H-representation with redundancies removed. Basis elements were normalized to unit length. The non-reduced individual k-cones, individual k-cones were calculated as M^−1^V, where M denotes the diagonal matrix with the mass action terms on its diagonal. The k-cones which where additionally constrained by equilibrium constants were calculated individually by adding the respective equilibrium constraints to the k-cone equation (see Supplementary Text). Visualization of the k-cones was performed by first performing dimensionality reduction using principal component analysis, followed by k-means clustering of the reduced vectors to avoid overlap for the k-cones not constrained by equilibrium constants. The convex hull, representing the shadow cast by the k-cone into the lower dimension, was calculated by identifying the set of non-redundant vectors in the reduced polytope.

The stability of the k-cone was obtained from calculations as detailed in Supplementary Text. Derivatives for the mass-action kinetics were derived analytically for each reaction and the Jacobian matrix constructed for each of the basis vectors of the corresponding flux cone. The stability for one basis vector was then evaluated based on the eigenvalues of the Jacobian matrices. The basis vector was identified as stable if all eigenvalues were smaller than −ε (where ε denotes the double float machine accuracy) and unstable if at least one eigenvalue was larger than ε.

### Optimization and differential analysis

Approximations of changes in enzyme activities were either obtained by the transformation T via calculating its log-diagonal log_2_ diag(T) = log_2_ (k_2_/k_1_) for T = M_1_M_2_^−1^, as derived in the Supplementary Text.

Necessity for proliferation was performed by first appending a biomass reaction to the model and identifying the maximum permissible flux through that reaction. The biomass reaction was adapted from Recon 2^60^. Here, we mapped metabolites or precursors from our model to the metabolites included in the Recon 2 model (version 2.02, also see Supplementary Text). In the case that one precursor could produce several metabolites in Recon 2, we used the maximum stoichiometry of the associated products. Given the resulting biomass reaction flux v_bm_, this procedure gave rise to the following linear programming program for the flux balance analysis^18^ of the fluxes v_i_:


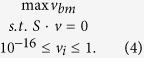


Here, the upper bound could be chosen arbitrarily, as later calculations used only the flux ratios, which are invariant to the upper bound. Lower bounds for the fluxes were chosen as 10^−16^ to ensure a non-zero flux for each reaction, as all reactions of the central carbon metabolism should be active in the cell lines used.

After obtaining the maximum biomass flux v_max_, the upper and lower flux limits were obtained by solving two linear programming problems for each flux v_i_:


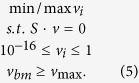


The largest absolute log-fold change that could be explained by flux variability analysis could now be obtained as


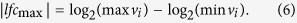


Finally, all individual log-fold changes obtained from the EDAs whose absolute value exceeded that of lfc_max_ were deemed necessary for proliferation.

All log-fold changes, obtained by the transformation T, were handled in the same way. Significance measures were obtained by performing Welch t-tests on the log-transformed mass-action terms after validating their normal distributions (Fig. S1). Sample variances were estimated using the empirical Bayes method as implemented in limma^36^,^37^. P-values obtained for all reactions were finally adjusted to q-values (false discovery cutoffs) by the method of Benjamini-Hochberg^61^. As an alternative to hypothesis testing, we also estimated log-fold changes by a combinatorial method. First, control log-fold changes were obtained from all permutations of HaCaT samples, yielding 6 control log-fold changes for each reaction with a zero mean. Log-fold changes were also obtained for all combinations of HeLa samples with a HaCaT samples, yielding 9 differential but possibly interdependent log-fold changes. Those combinatorial estimates were then used to obtain 95% credible intervals using the Bayes bootstrap^62^. The 95% credible intervals denote the one interval which contains the true log-fold change with 95% probability. The obtained p-values, as well as the mean log-fold, worst-case estimates, 95% credible intervals and standard deviations for the EDAs are reported in Table S3.

### Gene expression and coregulation

Fifty-eight microarray samples were manually selected from the GEO database by selecting for samples from a single platform (HGU133Plus 2.0), in untreated conditions and only for the previously used cell lines (HaCaT, keratinocytes and HeLa). The analysis of gene expression was performed by normalizing the raw data for the 58 samples by Frozen Robust Multiarray Analysis (fRMA), followed by differential analysis using the limma package, particularly its empirical Bayes method^36^,^63^. The exact protocol for the analysis can again be found in the Supplementary Text.

Standard deviations for log-fold changes of enzyme activities were obtained from the 9 possible Hela/HaCaT sample combinations of log-fold changes as used in the approximation of credible intervals. To obtain an estimate for the standard deviation in untreated conditions we used the control samples obtained from the paired permutations of HaCaT samples, as previously described. Given the control standard deviation σ_c_ and the differential standard deviation σ_d_ (obtained from the log-fold change samples described before), a reaction was considered differentially heterogeneous if σ_d_/σ_c_ > 3, based on the visualization in Fig. 5B that showed that the majority of reactions fell into that margin. Correlation between the selected heterogeneous enzymes was calculated by Pearson correlation between the corresponding 9 HeLa/HaCaT log-fold changes.

## Additional Information

**How to cite this article**: Diener, C. *et al*. The space of enzyme regulation in HeLa cells can be inferred from its intracellular metabolome. *Sci. Rep.* **6**, 28415; doi: 10.1038/srep28415 (2016).

## Supplementary Material

Supplementary Information

Supplementary Dataset 1
