# Supplementary material for: The space of enzyme regulation in HeLa cells can be inferred from its intracellular metabolome
Source: Sci Rep. 2016 Jun 23;6:28415. doi: 10.1038/srep28415 (PMC4917846; doi:10.1038/srep28415)
Supplement: Supplementary Information [file srep28415-s1.pdf]

Supplementary Text

The space of enzyme regulation in HeLa cells can be inferred from its intracellular metabolome

Christian Diener, Felipe Muñoz-Gonzalez, Sergio Encarnación, Osbaldo Resendis-Antonio

This document includes:

1. Supplementary Figures S1 to S3
2. Mathematical derivation of the used formalism
3. Rmarkdown protocol to reproduce the results of the paper

Figure S1

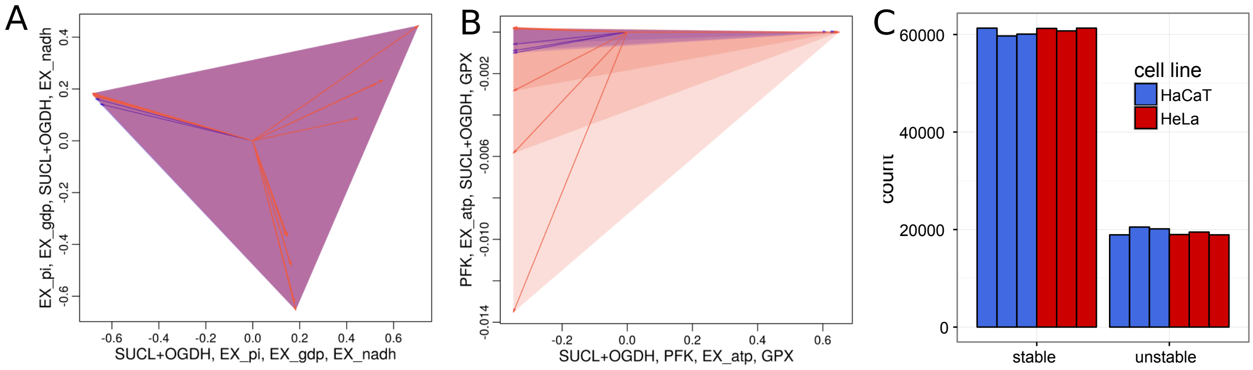

Supplementary Figure S1 - Additional analyses of k-cone spaces. (A) The k-cone reduced by adding additional constraints obtained from approximate *in vivo* equilibrium constants. Information captured in the reduction is 28.7%. (B) The k-cone constrained by the approximate equilibrium constants when only selecting reactions that change at least 2-fold. Information captured in the reduction is 99.9%. Equilibrium constants where obtained from `http://equilibrator.weizmann.ac.il/` assuming a pH of 7.34 and an ionic strength of 0.15 M for all samples. (C) Bars denote the number of basis vectors in the respective non-reduced k-cone that lead to stable or unstable steady states for the two used cell lines.

Figure S2

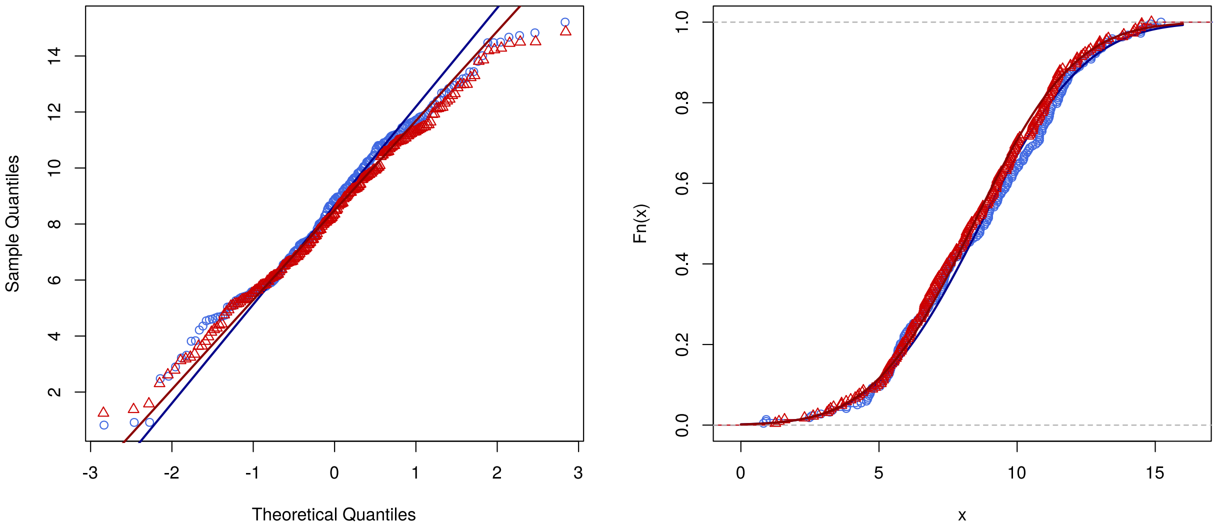

Supplementary Figure S2 - Metabolite concentrations are log-normal distributed The distribution of the log-transformed metabolite concentrations was compared to a normal distribution by quantile-quantile plots and empirical distribution functions. Blue symbols and lines denote the HaCaT samples and red symbols and lines the HeLa samples. Quantile-quantile plots are shown on the left and a perfect fit is denoted by the straight lines. Decent agreement is observed, with some deviations in the tails. The empirical distribution functions are shown on the right, and cumulative distribution functions for a normal distribution is indicated by the straight lines.

Figure S3

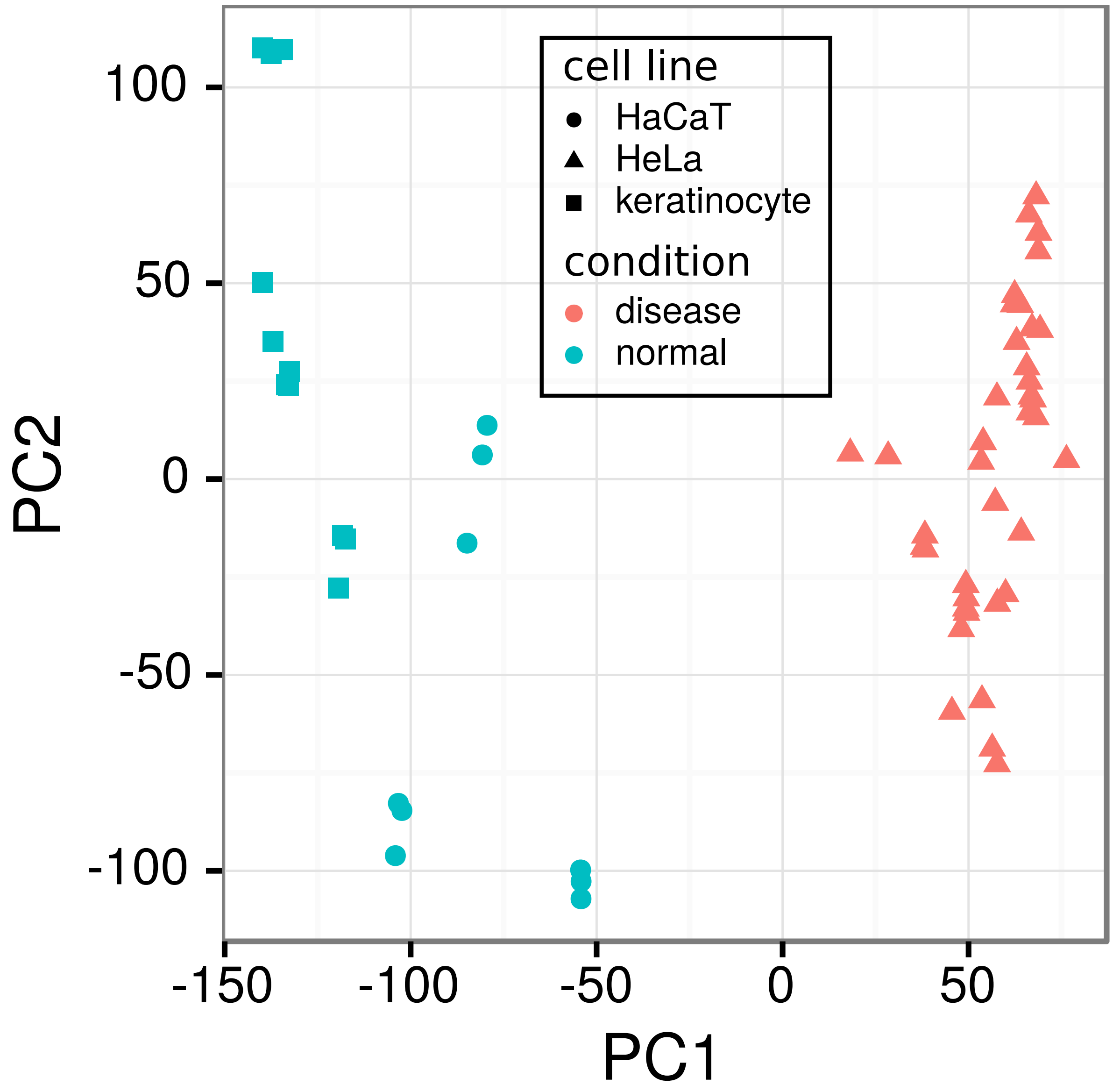

Supplementary Figure S3 - Mapping of microarray samples to the first two principal components. Colors denote condition, and shape denotes cell lines. The samples separate well into non-cancerous normal samples and cancerous HeLa samples. The total standard deviation contained in the first two principal components was 17.6%.

Mathematical description of the k-cone formalism

1 The flux cone

Given the stoichiometric matrix $\mathbf{S}$[1] and the vector of reaction velocities $\mathbf{v} = \mathbf{v}(\mathbf{S}, t)$ the steady state condition is given by

$$\mathbf{S} \cdot \mathbf{v} = \mathbf{0}. \tag{1}$$

The fact that some reactions might be irreversible enforces positivity constraints on some elements of $\mathbf{v}$ (the irreversible reactions). In order to make the constraints more consistent one often splits up all reversible reactions into their respective forward and backward reactions. This can be achieved by a link matrix $\mathbf{L}$ which has a block diagonal shape with ones on the diagonal for irreversible reactions and $(1, -1)$ for reversible reactions. For instance for the simple system

$$\xrightarrow{v_1} A \underset{v_2}{\overset{v_3}{\rightleftarrows}} B \text{ with } \mathbf{S}_{rev} = \begin{pmatrix} 1 & -1 \\ 0 & 1 \end{pmatrix} \tag{2}$$

we can formulate the following transformation to get the irreversible stoichiometric matrix $\mathbf{S}_{irr}$:

$$\mathbf{S}_{irr} = \begin{pmatrix} 1 & -1 & 1 \\ 0 & 1 & -1 \end{pmatrix} = \mathbf{S}_{rev} \cdot \mathbf{L} = \begin{pmatrix} 1 & -1 \\ 0 & 1 \end{pmatrix} \cdot \begin{pmatrix} 1 & 0 & 0 \\ 0 & 1 & -1 \end{pmatrix}. \tag{3}$$

Using the link matrix $\mathbf{L}$ we can now formulate our steady state condition for the irreversible stoichiometric matrix $\mathbf{S}_{irr}$ and split fluxes $\mathbf{v}_{irr}$

$$\mathbf{S}_{irr}\mathbf{v}_{irr} = \mathbf{S}_{rev}\mathbf{L}\mathbf{v}_{irr} = \mathbf{0}. \tag{4}$$

This system now has a semi-trivial solution $\mathbf{L}\mathbf{v}_{irr}$ which requires irreversible reactions to carry zero flux and reversible reactions to be in chemical equilibrium, meaning that the respective forward and backward reactions carry the same flux. However, as long as $\mathbf{S}_{rev}$ is invertible there may

[1] In this text all lower-case bold letters denote vectors, all upper-case bold letters matrices and all cursive letters denote scalars. At the same time the multiplictation operator, "·", denotes scalar multiplication for scalars, the dot product for vectors and matrix multiplication for matrices. For conciseness the multiplication operator will sometimes be omitted.

also be other non-equilibrium solutions for the steady state.

From here on $\mathbf{S}$ will always denote the irreversible stoichiometric matrix $\mathbf{S}_{irr}$ and $\mathbf{v}$ the irreversible fluxes $\mathbf{v}_{irr}$, yielding the steady state condition

$$\mathbf{S} \cdot \mathbf{v} = \mathbf{0} \text{ where } \forall i : v_i > 0 \tag{5}$$

This defines a solution space which is a pointed cone[2]. The basis for this space, $\mathbf{V}$, is a $m \times n$ matrix called the *flux cone* where

$$\forall \mathbf{v} = \mathbf{Vc} \text{ if } \mathbf{c} \in \mathbb{R}^{m,+} : \mathbf{Sv} = \mathbf{0}. \tag{6}$$

2 The k-cone

In the k-cone formalism we assume that, given the metabolite concentrations $\mathbf{x}$, the reaction velocities can be written as

$$v_j(\mathbf{x}) = k_j \cdot m_j(\mathbf{x}). \tag{7}$$

Here k_j denotes the reaction rate and m_j are metabolic terms, in particular, for mass-action kinetics it holds that $m_j = \prod_i x_i^{s_{ij}}$, where s_{ij} is equal to the stoichiometry of metabolite x_i in reation j. However, m_j terms can be chosen arbitrary in the k-cone formalism to acomodate other kinetics. m_j denotes the corresponding mass-action terms. Defining the diagonal matrix $\mathbf{M} := \text{diag}(m_j)$, one can rewrite the steady state condition as

$$\mathbf{SMk} = \mathbf{0}. \tag{8}$$

For a given vector of substrate concentrations $\mathbf{x}$, $\mathbf{SM}$ defines a convex polytope of all possible reaction rates, and thus enzyme activities, for the given steady state $\mathbf{X}$. Solving the vertex enumeration problem for the H-representation given by $\mathbf{SM}$ and the constraints on $\mathbf{k}$ defines a finite basis $\mathbf{K}$, called the *k-cone*. One could solve the vertex enumeration for a k-cone directly, however it is more practical to derive the k-cone directly from the flux cone $\mathbf{V}$ (as defined in eq. 6). Using the definition of the reaction velocities (equation 7) one can see that the k-cone $\mathbf{K}$ can be obtained by

$$\mathbf{K} = \mathbf{M}^{-1}\mathbf{V} \tag{9}$$

This requires the diagonal matrix $\mathbf{M}$ to be invertible, which is the case if all metabolic terms are none-zero. In practice zero metabolic terms denote reactions which are inactive and can be removed from the stoichiometric matrix. This defines one unique k-cone built from a skeleton flux cone, which gives a good basis for comparative analysis.

One should note that the k-cone can be constrained even further if one has measurements for *in vivo* equilibrium constants K_{eq}^i for a reversible reaction with index i, since it defines an equality

[2]meaning that the origin is included in the space and that all other points in the space are strictly positive

constraint for the forward and backward rate constants k_i^+ and k_i^- which may reduce the k-cone space:

$$K_{eq}^i = \frac{k_i^-}{k_i^+} \tag{10}$$

$$K_{eq}^i \cdot k_i^+ = k_i^- . \tag{11}$$

3 Conditional transitions

If we observe the same biochemical systems in two different conditions, normal (n) and disease (d), this defines their respective k-cones $\mathbf{K}_n$ and $\mathbf{K}_d$. Using equation 9 we can easily derive a transformation matrix $\mathbf{T}$ that describes the transition from the healthy to the disease space by the following theorem.

Theorem 1. *There exists a diagonal transformation* $\mathbf{T} = \mathbf{M}_n\mathbf{M}_d^{-1}$ *such that* $\mathbf{K}_d = \mathbf{T}\mathbf{K}_n$.

Proof. Directly follows by applying equation 9 to $\mathbf{K}_d$.

$$\mathbf{K}_d = \mathbf{M}_d^{-1}\mathbf{V} \tag{12}$$

$$= \mathbf{M}_d^{-1}\mathbf{M}_n\mathbf{K}_n \tag{13}$$

$$= \mathbf{M}_n\mathbf{M}_d^{-1}\mathbf{K}_n = \mathbf{T}\mathbf{K}_n \tag{14}$$

□

Thus, the transformation between the k-cone spaces is given by metabolome data alone. However, this is a property of the entire spaces. Within the respective spaces the kinetic constants can assume any value within the respective k-cone.
However, this requires the solutions to come from the same flux cone $\mathbf{V}$ which is only the case if the stoichiometric matrix $\mathbf{S}$ and constraints are the same in the normal and disease condition. Thus, we will now aim at deriving a more general expression.

We will first formulate equation 7 in a vector form by considering the (irreversible) fluxes $\mathbf{v}$ given by

$$\mathbf{v} = \mathbf{M} \cdot \mathbf{k}. \tag{15}$$

Hence, if we observe the same biochemical systems in two different conditions, normal (n) and disease (d), the respective enzyme activities are given by

$$\mathbf{k}_n = \mathbf{M}_n^{-1}\mathbf{v}_n \text{ and} \tag{16}$$

$$\mathbf{k}_d = \mathbf{M}_d^{-1}\mathbf{v}_d. \tag{17}$$

This raises the question if there exists an affine transformation between the normal and disease state.

Theorem 2. *Given enzyme activities* $\mathbf{k}_n$ *and* $\mathbf{k}_d$ *it holds that*

$$\mathbf{k}_d = \mathbf{T}\mathbf{W}\mathbf{k}_n \text{ where} \tag{18}$$
$$\mathbf{T} = \mathbf{M}_n\mathbf{M}_d^{-1} \tag{19}$$
$$\mathbf{W} = \operatorname{diag}(\mathbf{v}_d/\mathbf{v}_n). \tag{20}$$

Proof. Straight forward using equations 17 and the commutativity of diagonal matrices.

$$\begin{aligned}
\mathbf{k}_d &= \mathbf{M}_d^{-1}\mathbf{v}_d && (21)\\
&= \mathbf{M}_d^{-1}\operatorname{diag}(\mathbf{v}_d/\mathbf{v}_n)\mathbf{v}_n && (22)\\
&= \mathbf{M}_d^{-1}\operatorname{diag}(\mathbf{v}_d/\mathbf{v}_n)\mathbf{M}_n\mathbf{M}_n^{-1}\mathbf{v}_n && (23)\\
&= \mathbf{M}_n\mathbf{M}_d^{-1}\operatorname{diag}(\mathbf{v}_d/\mathbf{v}_n)\mathbf{M}_n^{-1}\mathbf{v}_n && (24)\\
&= \mathbf{T}\mathbf{W}\mathbf{M}_n^{-1}\mathbf{v}_n = \mathbf{T}\mathbf{W}\mathbf{k}_n && (25)
\end{aligned}$$

□

This gives an affine transformation between the enzyme activities given by the normal and disease state. Furthermore, the transition is composed of the transformation matrix $\mathbf{T}$ and the weight matrix $\mathbf{W}$. Even though the theorem did not assume the same flux cone for both conditions this time, it recovers the transformation matrix $\mathbf{T}$ again. Since $\mathbf{T}$ and $\mathbf{W}$ are diagonal one can intepret their product as weighting each diagonal entry in $\mathbf{T}$ by the diagonal entries in $\mathbf{W}$. Thus, from a biochemical point of view, the change in enzyme activities from the normal to the disease state can be decomposed into changes in the mass-action terms (the concentration-dependent part of the kinetics) and the flux changes between the two conditions. $\mathbf{W}$ is usually not known, but $\mathbf{T}$ can easily be calculated from steady state measurements of metabolites. A healthy system using $\mathbf{k}_n$ must traverse through $\mathbf{TW}$ towards $\mathbf{k}_d$. Thus, $\mathbf{TW}$ contains all possible changes to traverse into the disease state and acts as a representation for the regulatory events occurring between $\mathbf{k}_n$ and $\mathbf{k}_d$. Reactions with large entries in $\mathbf{T}$ are either regulated or must show a strong alteration in their steady state flux balance, given by $\mathbf{v}_d/\mathbf{v}_n$, that counteracts the entries in $\mathbf{T}$. The more similar the steady state flux distributions of the normal and disease state are, the more meaningful is $\mathbf{T}$. In particular, if the flux cones $\mathbf{V}_n$ and $\mathbf{V}_d$ are similar, randomly sampling feasible fluxes $\mathbf{v}_n, \mathbf{v}_d$ would in average result in a weight matrix $\mathbf{W}$ close to the identity matrix and $\mathbf{TW} \approx \mathbf{T}$.

4 Stability analysis

The dynamics of the system are given by the ODE system

$$\frac{d\mathbf{x}}{dt} = \mathbf{S}\mathbf{v}(\mathbf{x}) = \mathbf{S}\mathbf{M}(\mathbf{x})\mathbf{k} \tag{26}$$

Evaluating this ODE system at a perturbed steady state $\mathbf{x}^* + \Delta\mathbf{x}$ and performing a linear ap-

proximation yields

$$\frac{d\mathbf{x}^* + \mathbf{x}}{dt} = \mathbf{SM}(\mathbf{x}^* + \Delta\mathbf{x})\mathbf{k} \tag{27}$$

$$0 + \frac{d\mathbf{x}}{dt} \approx \mathbf{S}\,\mathbf{diag}(\mathbf{k})\mathcal{J}(\mathbf{x}^*)\Delta\mathbf{x}. \tag{28}$$

Here $\mathcal{J}(\mathbf{x}^*)$ denotes the Jacobian for the metabolic terms with $\mathcal{J}(\mathbf{x}) = \left(\frac{dm_j(\mathbf{x})}{d\mathbf{x}_i}\right)_{ij}$. The solution to this system is given by the matrix exponential via

$$\Delta\mathbf{x}(t) = \exp(\mathbf{S}\,\mathbf{diag}(\mathbf{k})\mathcal{J}(\mathbf{x}^*)t)\Delta\mathbf{x}(0). \tag{29}$$

Given a k-cone $\mathbf{K} = \{\mathbf{k_i}\}$ we can use its basis property to express all possible $\mathbf{k}$ by $\mathbf{k} = \sum_i w_i\mathbf{k}_i$ ($w_i \geq 0 \in \mathbb{R}$), yielding

$$\Delta\mathbf{x}(t) = \exp(\mathbf{S}\,\mathbf{diag}(\sum_i w_i\mathbf{k}_i)\mathcal{J}(\mathbf{x}^*)t)\Delta\mathbf{x}(0) \tag{30}$$

$$= \prod_i \exp(w_i\mathbf{S}\,\mathbf{diag}(\mathbf{k}_i)\mathcal{J}(\mathbf{x}^*)t)\Delta\mathbf{x}(0) \tag{31}$$

The matrix exponentials are given by the eigendecomposition, resulting in

$$\Delta\mathbf{x}(t) = \prod_i \mathbf{Q}_i \exp(w_i\mathbf{\Lambda}_i t)\mathbf{Q}_i^{-1}\Delta\mathbf{x}(0) \tag{32}$$

where $\mathbf{Q}_i$ contains the eigenvectors pertaining[3] to $w_i\mathbf{k}_i$ and $\mathbf{\Lambda}_i$ its eigenvalues on the diagonal. Each elements of the solution is, thus, denoted by a reweighted product of exponentials

$$\Delta x_i(t) = \alpha_i \exp(\sum_{i,j} \phi_{ij}\lambda_{ij}t)\Delta x_i(0) \tag{33}$$

for some weights α_i and indices i, j with associated weights $\phi_{i,j}$.

Thus, some conclusions about the stability of a solution can be drawn based on the eigenvalues pertaining to the basis vectors in $\mathbf{K}$. Particularly, if $w_i > 0$ only for stable basis vectors ($\Re(\mathbf{\Lambda}_i) < 0$) all resulting $\mathbf{k}$ will lead to a steady state as well. In case some of the basis vectors are unstable no general conclusion can be drawn since, due to the product in the expression, the resulting system can either be stable or unstable based on the structure of the matrices $\mathbf{Q}_i$ and the weights w_i. However, due to the sum form in equation 33 one can see that the magnitude of the eigenvalues dictate the resulting stability, meaning that large absolute eigenvalues from a stable state can dominate small unstable ones.

[3]"pertaining" meaning that $\mathbf{Q}_i$ contains the eigenvectors of $w_i\mathbf{S}\,\mathbf{diag}(\mathbf{k}_i)\mathcal{J}(\mathbf{x}^*)$ and $\mathbf{\Lambda}_i$ its eigenvalues on the diagonal.

k-cone analysis protocol

Protocol: k-cone analysis of HeLa cells

Installation

All of the analysis is performed in R. As such the first thing you will need is to install R. For installation instructions see http://r-project.org. In Ubuntu and Debian R can be installed via the Terminal using

```
sudo apt-get install r-base r-base-dev
```

Additionally some of the dependencies of `dycone` require development versions of some libraries for web security and scraping. In Ubuntu and Debian those can be installed via

```
sudo apt-get install libxml2-dev libcurl4-openssl-dev libssl-dev libgmp-dev
```

Most of the actual analysis is implemented in the `dycone` R package. It can be installed using devtools in the following manner. We will also install all optional dependencies so we can build this document. In a Terminal type `R` to start R, than use the following commands:

```
install.packages("devtools")
source("http://bioconductor.org/biocLite.R")
biocLite(c("Biobase", "IRanges", "AnnotationDbi", "affy", "frma", "genefilter",
    "GEOquery", "hgu133plus2.db", "hgu133plus2frmavecs", "limma"))
devtools::install_github("cdiener/dycone", dependencies = TRUE)
```

This will install dycone and all additional dependencies. You will see at lot of messages from the compiler and the whole process might take a few minutes. After that the dycone library can be loaded with

```
library(dycone)
```

Reading the model and additional data

Dycone models can be obtained by a variety since for most analysis an irreversible stoichiometric matrix is sufficient. However `dycone` uses an internal representation which is a list of reactions. Here each list entry requires at least the entries `S`, `P`, `N_S`, `N_P` and `reversible` (all vectors) specifying the names of substrates and products, the respective stoichiometries and the reversibility of the reaction. Each reaction can furthermore carry an arbitrary amount of annotations. Those models can be read from a csv-like file format. The model used in this analysis can be found in "reactions.csv" and be read and output easily by

```
library(dycone)

r <- read_reactions("reactions.csv")
print(r)
```

```
## Model has 59 reactions (41 reversible)
## BPGM:  1*13dpg <=> 1*23dpg
## BPGM:  1*23dpg -> 1*3pg + 1*pi
```

```
## ENO:  1*2pg <=> 1*pep
## ALDO:  1*fdp <=> 1*dhap + 1*g3p
## FBP:  1*fdp -> 1*f6p + 1*pi
## GAPDH:  1*g3p + 1*nad + 1*pi <=> 1*13dpg + 1*nadh
## PFK:  1*atp + 1*f6p -> 1*adp + 1*fdp
## GPI:  1*g6p <=> 1*f6p
## ACYP:  1*13dpg -> 1*pi + 1*3pg
## PGK:  1*3pg + 1*atp <=> 1*13dpg + 1*adp
## PGAM:  1*2pg <=> 1*3pg
## PGM:  1*g1p <=> 1*g6p
## PKM:  1*adp + 1*pep -> 1*atp + 1*pyr
## TPI:  1*dhap <=> 1*g3p
## LDH:  1*nad + 1*lac-L <=> 1*nadh + 1*pyr
## G6PD:  1*g6p + 1*nadp -> 1*6pgl + 1*nadph
## PGD:  1*nadp + 1*6pgc <=> 1*nadph + 1*ru5p-D
## PGL:  1*6pgl -> 1*6pgc
## PGM:  1*r1p <=> 1*r5p
## PRPS:  1*atp + 1*r5p <=> 1*amp + 1*prpp
## RPE:  1*ru5p-D <=> 1*xu5p-D
## RPI:  1*r5p <=> 1*ru5p-D
## TALDO:  1*g3p + 1*s7p <=> 1*f6p + 1*e4p
## TKT:  1*r5p + 1*xu5p-D <=> 1*g3p + 1*s7p
## TKT:  1*xu5p-D + 1*e4p <=> 1*f6p + 1*g3p
## ACO:  1*cit <=> 1*icit
## PDH+CS:  1*oaa + 1*pyr + 1*nad -> 1*cit + 1*nadh
## FH:  1*fum <=> 1*mal-L
## ICDH3:  1*nad + 1*icit <=> 1*nadh + 1*akg
## ICDH1:  1*icit + 1*nadp <=> 1*akg + 1*nadph
## MDH:  1*nad + 1*mal-L <=> 1*nadh + 1*oaa
## SDH:  1*succ <=> 1*fum
## SUCL+OGDH:  1*succ + 1*atp + 1*nadh <=> 1*akg + 1*nad + 1*adp + 1*pi
## SUCL+OGDH:  1*succ + 1*gtp + 1*nadh <=> 1*akg + 1*nad + 1*gdp + 1*pi
## ME1:  1*mal-L + 1*nad <=> 1*pyr + 1*nadh
## ME2:  1*mal-L + 1*nadp <=> 1*pyr + 1*nadph
## PCK:  1*oaa + 1*gtp <=> 1*pep + 1*gdp
## PC:  1*pyr + 1*atp -> 1*oaa + 1*adp + 1*pi
## GLS:  1*gln <=> 1*glu
## GLUD:  1*glu + 1*nad <=> 1*akg + 1*nadh
## GLUD:  1*glu + 1*nadp <=> 1*akg + 1*nadph
## HA:  1*pi + 1*adp -> 1*atp
## ND:  1*nadh -> 1*nad
## GPX:  2*gsh -> 1*gssg
## GSR:  1*gssg + 1*nadph -> 2*gsh + 1*nadp
## EX_g1p:   -> 1*g1p
## EX_gln:   <=> 1*gln
## EX_nadp:   <=> 1*nadp
## EX_nadph:   <=> 1*nadph
## EX_adp:   -> 1*adp
## EX_amp:   <=> 1*amp
## EX_nad:  1*nad ->
## EX_nadh:   -> 1*nadh
## EX_pi:   <=> 1*pi
## EX_gdp:   <=> 1*gdp
## EX_gtp:   <=> 1*gtp
```

```
## EX_gsh:   <=> 1*gsh
## EX_atp:  1*atp ->
## EX_lac:  1*lac-L <=>
```

Looking at the first reaction we see the additional annotations

```r
r[[1]]
```

```
## $S
## [1] "13dpg"
##
## $P
## [1] "23dpg"
##
## $N_S
## [1] 1
##
## $N_P
## [1] 1
##
## $rev
## [1] TRUE
##
## $abbreviation
## [1] "BPGM"
##
## $pathway
## [1] "Glycolysis"
##
## $KEGG_reaction
## [1] "R01662"
##
## $KEGG_enzyme
## [1] "5.4.2.4"  "5.4.2.11"
##
## $Keq
## [1] 7300000
```

The metabolome measurements are found in in "metabolome.csv" (Table S1 in the manuscript). Here "Rx" denotes repetition x and "He/Ha" the HeLa and HaCaT cell lines. The measurements are given as pmol per million cells. First we will convert those to micro-mole per liter by using the experimentally quantified volume of HeLa cells (1.54 fL, http://doi.org/10.1002/nbm.1173).

```r
metab <- read.csv("metabolome.csv")
# First 4 columns are annotations
metab[, 5:10] <- 1e-6 * metab[, 5:10]/(1e6 * 1.54e-12)
```

As one can see, the names used by the provider during metabolite measurements are not the same we used in the model. Thus, we will also need an ID map to identify the metabolites. This map is given in `id_map.csv`

```r
id_map <- read.csv("id_map.csv", stringsAsFactors=FALSE)
head(id_map)
```

```
##    name            kegg                  hmdb
## 1 13dpg          C00236            HMDB01270
## 2   3pg C00197, C00597  HMDB60180, HMDB00807
## 3    pi          C00009 HMDB01429, HMDB02142
## 4 23dpg          C01159            HMDB01294
## 5   2pg          C00631            HMDB03391
## 6   pep          C00074            HMDB00263
```

Some of the metabolites have several IDs assigned to them. We will come back to that later.

The log-fold changes for all combinations between HeLa and HaCaT cells can be obtained by

```
library(tidyr)
library(ggplot2)

comb <- expand.grid(a = 1:3, b = 4:6)
mlfc <- apply(metab[, 5:10], 1, function(m) log(m[comb$b], 2) - log(m[comb$a], 2))
colnames(mlfc) <- as.character(metab$name)
mlfc <- gather(data.frame(mlfc, check.names = F), name, lfc, factor_key=T)
ggplot(mlfc, aes(x = name, y = lfc)) + geom_boxplot() + theme_bw() +
    theme(axis.text.x = element_text(angle = 90, hjust = 1, vjust = 0.5)) +
    xlab("") + ylab("log-fold change")
```

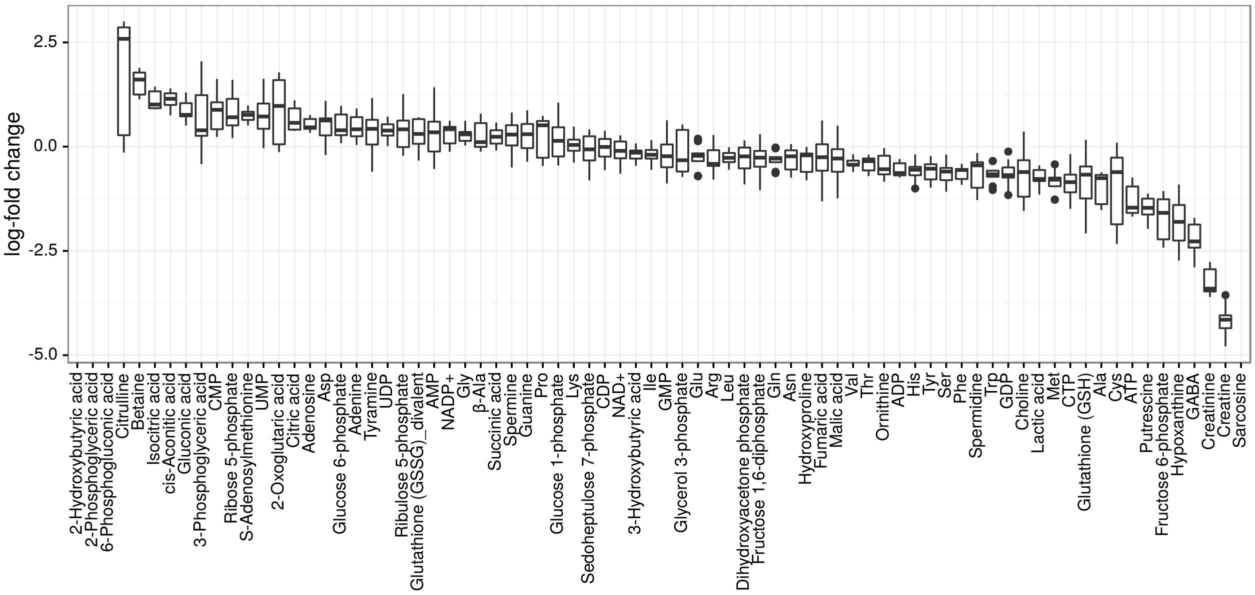

Imputation of missing data

At first we will try to see which metabolites in the model are missing in the measurements. For this we will look for matches of the respective metabolites from the model in the metabolome measurements. Matching will be done based on KEGG IDs.

```
matches <- sapply(id_map$kegg, grep_id, x = metab$kegg_id)
miss <- is.na(matches)
table(miss)
```

```
## miss
## FALSE  TRUE
##    28    15
```

So we see we have measurements for about 2/3 of the required metabolites To impute the missing values we will first construct a data frame for all metbaolites with missing entries and see how to fill it in the following steps.

```
full <- id_map[, 1:2]
d_idx <- 5:10
m <- matrix(NA, nrow = nrow(full), ncol = length(d_idx))
colnames(m) <- names(metab)[d_idx]
full <- cbind(full, m)
matched_idx <- !is.na(matches)
full[matched_idx, 3:8] <- metab[matches[matched_idx], d_idx]
```

`full` now already includes the metabolome measurements but still has missing entries which we will now scrape from the Human Metabolome Database (HMDB, http://hmdb.ca). HMDB assigns a single KEGG is to each metabolite in the database. However, there are cases where a single metabolite can be identified by several IDs in the HMDB. This may happen if we have "Glucose" which may for instance map to "alpha-D-Glucose" or "beta-D-Glucose". This is the reason why our `id_map` includes several HMDB and KEGG IDs for some metabolites.

We will now scrape the concentrations for all metabolites in the model from HMDB. This might take some time so we will use the caching operator `%c%` from dycone which will take an R expression and will cache all assigned variables in that expression to a cache file, so that rerunning the script will read the results from the cache and not rerun the analysis. To run the analysis again simply delete the cache file without changing any of the code.

```
{
    concs <- hmdb_concentration(id_map$hmdb, add = id_map[, 1:2])
} %c% "scraped_concs.Rd"
```

We use this data to get the mean values for measured concentrations (HMDB quantifies almost all metabolites by micro-moles per liter as well) by taking them from cytoplasm measurements where available, or blood as a fallback. (In any way those imputations will only be used for stability analysis and not for differential measurements. If one only wants to use the differential analysis of `dycone` you can simply substitute all NA values in `full` by any constant.)

```
m_concs <- as.vector(by(concs, concs$name, priority_mean))
names(m_concs) <- levels(factor(concs$name))
```

In order to fill the gaps in the `full` data set we will use the `patch` function from dycone. `patch` will first to attempt to fill any hole with measurements from the own data set, either by using the mean values of the same cell line or, if not available, by the mean value of the other cell line. Thus, giving priority to the local data before the scraped ones. Only measurements with missing entries in both cell lines are filled with the scraped mean concentrations from HMDB.

```
scraped <- data.frame(kegg = names(m_concs), normal = m_concs)
rownames(scraped) <- NULL
patched <- patch(full, id = 1, normal = 3:5, treatment = 6:8, ref_data = scraped)
head(patched)
```

```
##    name            kegg       R1Ha       R2Ha       R3Ha       R1He
## 1 13dpg          C00236    0.40000    0.40000    0.40000    0.40000
## 2   3pg C00197, C00597   94.98857   59.30123   54.11558  223.56188
## 3    pi          C00009  633.75500  633.75500  633.75500  633.75500
## 4 23dpg          C01159 4500.00000 4500.00000 4500.00000 4500.00000
## 5   2pg          C00631   28.88023   28.88023   28.88023   40.14753
## 6   pep          C00074   17.00000   17.00000   17.00000   17.00000
##          R2He       R3He
## 1    0.40000    0.40000
## 2   74.10929   71.02831
## 3  633.75500  633.75500
## 4 4500.00000 4500.00000
## 5   17.61292   28.88023
## 6   17.00000   17.00000
```

This yields a complete data set `patched` which we will use for the dycone analysis.

The k-cone of HaCaT and HeLa cells

As described in more detail in the Supplementary Text of the publication the k-cone of several metabolome measurement can be generated from a skeleton flux cone. Thus, we will first calculate the flux cone for our model. This will take about half an hour, so we will use the caching operator again to avoid recalculating the flux cone every time we run the analysis. For this we will need an irreversible stoichiometric matrix which can be generated with the `stoichiometry` function of `dycone`.

```
S <- stoichiometry(r)
{ V <- polytope_basis(S) } %c% "basis.Rd"
dim(V)
```

```
## [1]   100 80231
```

The flux cone has >80.000 basis vectors here.

For the metabolic terms we use the mass-action terms generated from the imputed metabolome measurements. This is sufficient to create the k-cones for the six measurements. `ma_terms` expects a single named vector of concentrations, or a data frame with a "name" column and several columns containing concentrations.

```
mats <- ma_terms(S, patched[, c(1, 3:8)])
K <- lapply(1:6, function(i) kcone(V, mats[, i]))
# Reaction names to annotate the axis
rn <- rp(make_irreversible(r), "abbreviation")[,2]
```

To visualize the k-cone we will use the `plot_red` function which first projects the high-dimensional k-cone into the two dimension capturing the most variance, followed by clustering of the extreme rays of the cone to avoid repeatedly using rays that are very similar. The shaded area corresponds to the interior of the cone, so all feasible sets of kinetic constants fall into the shaded area of the respective k-cone. We will use blue for HaCaT cells and red for HeLa. Since the used k-means clustering is not entirely deterministic the images here might look a bit different here than in the publication (particularly arrow outliers) or be rotated along one of the two PC axes. However, the general appearance of the cones should be the same.

```r
plot_red(K, col = rep(c("blue", "tomato2"), each=3), r_names=rn)
```

```
## Bases are very large. Reducing to 201 clusters...
## Mean in-cluster distance: 0.12954.
## Information captured in projection: 93.365955%.
```

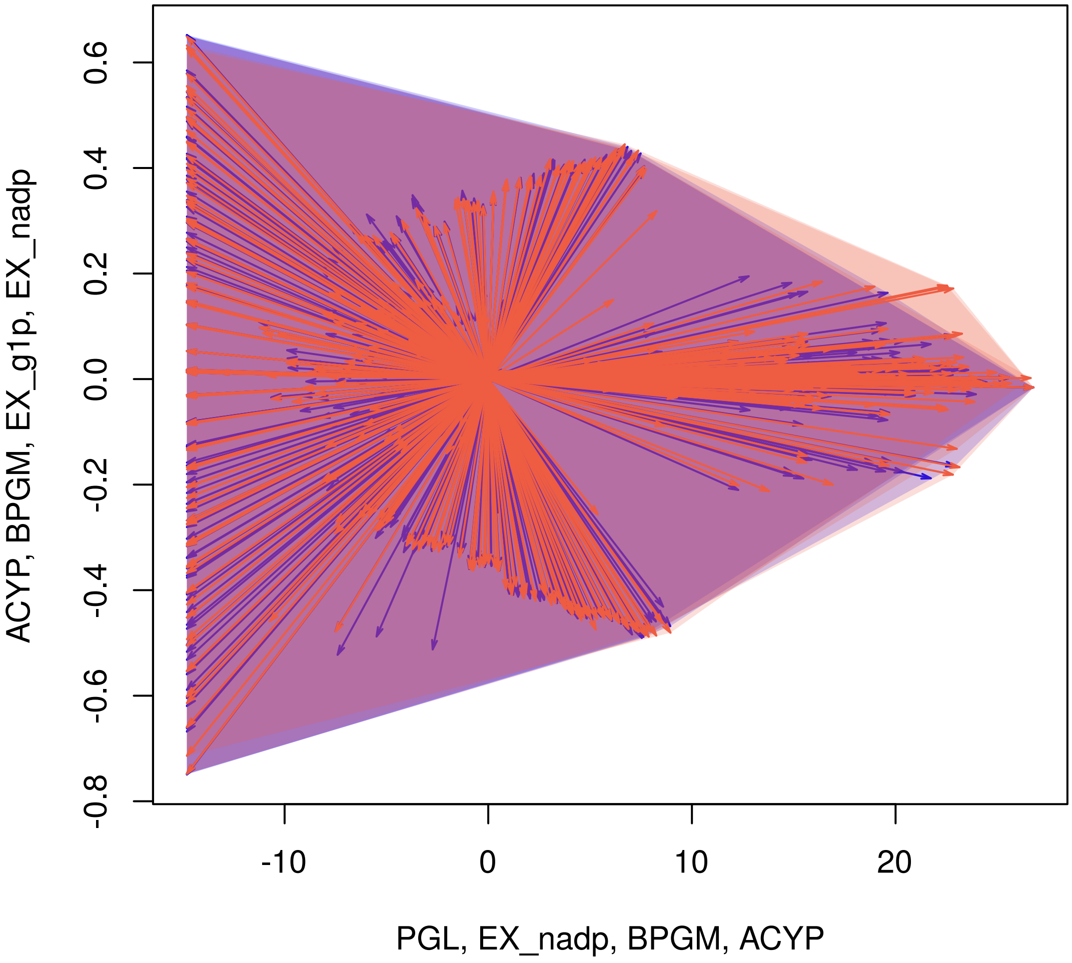

As we can see there are only minor differences between the k-cones. To see the proportion of the cone with large entries in the transformation matrices we will first calculate the log2-fold changes between all combinations of HeLa and HaCaT cells and only use those with an absolute log2-fold change larger than 1 (thus, a fold change larger than 2).

```r
combs <- expand.grid(4:6, 1:3)
lfcs <- apply(combs, 1, function(i) log(mats[, i[2]], 2) - log(mats[, i[1]], 2))
large <- abs(rowMeans(lfcs)) > 1
K_subset <- lapply(K, function(ki) ki[large, ])
plot_red(K_subset, col = rep(c("blue", "tomato2"), each=3), r_names=rn[large])
```

```
## Bases are very large. Reducing to 201 clusters...
## Mean in-cluster distance: 2.74911e-05.
## Information captured in projection: 93.184890%.
```

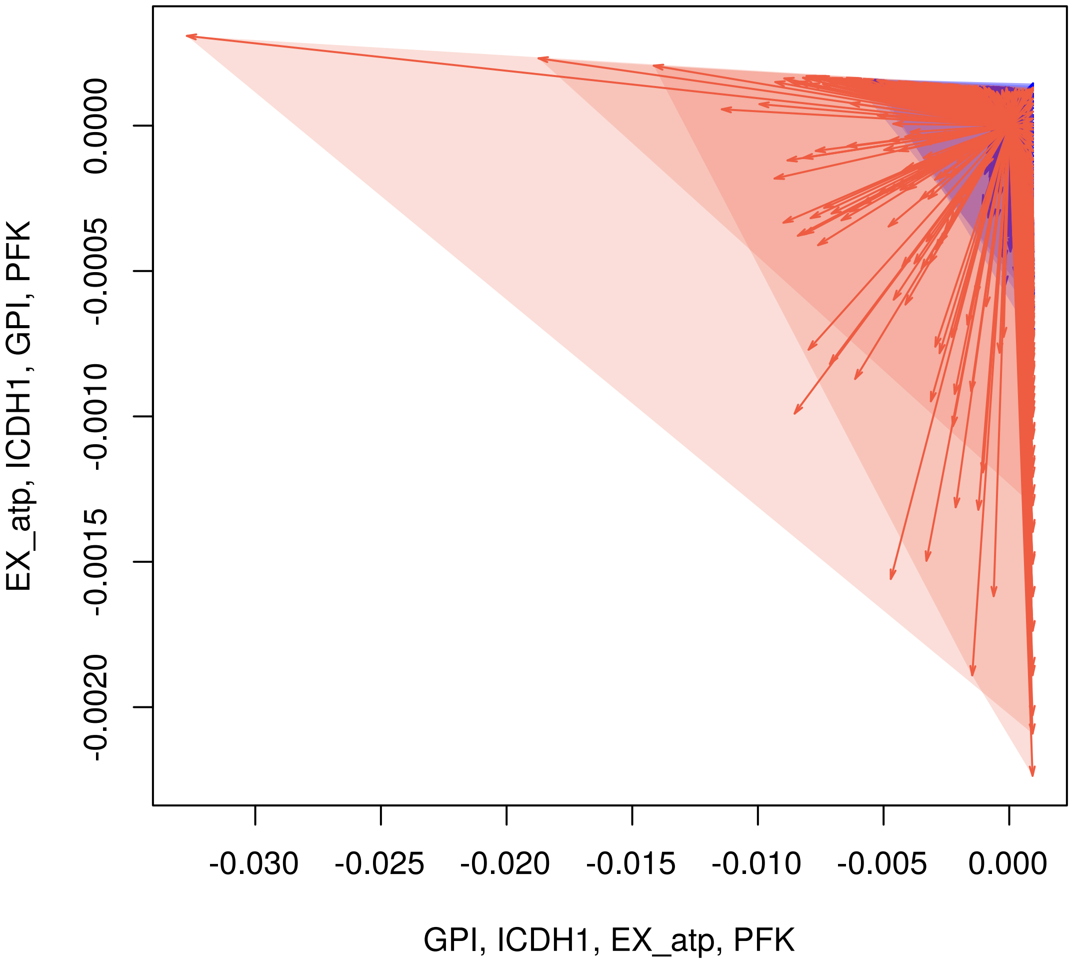

We can also reduce the k-cone spaces even further by using measurements for *in vivo* equilibrium constants Keq. Because there are no measured Keq for our cell lines we will use approximations obtained from http://equilibrator.weizmann.ac.il/. For that we will assume an pH of 7.34 and an ionic strength of 0.15 M for all samples. The estimated Keq values are already contained in the model. And we can thus use the `constrain_by_Keq` function from `dycone`. In order to accelerate this, we again use the caching operator and will also employ the `doParallel` package to run the analysis for each k-cone in parallel. If you have less than 6 CPU cores adjust the option accordingly or simply do not setup the cluster which will cause the code to run on a single core.

```
# this part is optional
library(doParallel)
registerDoParallel(cl = 6)
# end of optional part

{
    K_small <- foreach(i = 1:6) %dopar% {
        keq_constraints <- constrain_by_Keq(r)
        polytope_basis(S, zero_eq=keq_constraints, m_terms=mats[,i])
    }
} %c% "basis_keq.Rd"

par(mfrow=1:2)
plot_red(K_small, col = rep(c("blue", "tomato2"), each=3), r_names = rn)
```

```
## Information captured in projection: 28.727957%.
```

```
K_subset <- lapply(K_small, function(ki) ki[large, ])
plot_red(K_subset, col = rep(c("blue", "tomato2"), each=3), r_names = rn[large])
```

```
## Information captured in projection: 99.907257%.
```

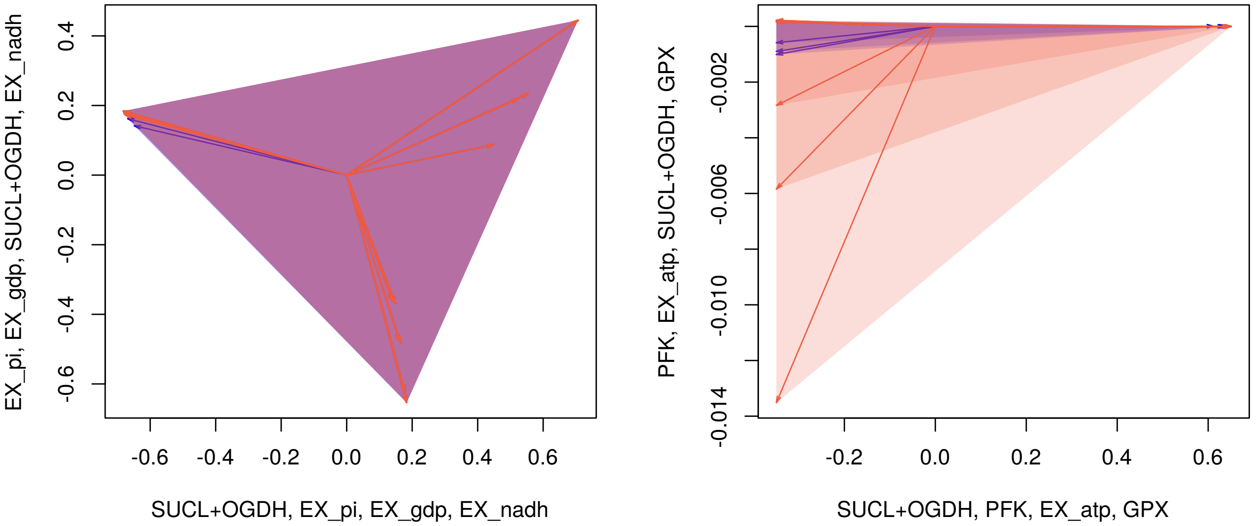

Stability analysis

To get the stability for an entire k-cone we need to run a stability analysis for each basis vector of the k-cone. Since we have 6 k-cones with over 80.000 basis vectors each this will take a while so we will perform it in parallel again.

```
{
    stab <- foreach(i = 1:6, .combine = rbind) %dopar% {
        concs <- patched[, 2 + i]
        names(concs) <- patched$name
        s <- stability_analysis(kcone(V, mats[, i]), S, concs)$what
        data.frame(what = s, basis_idx = i)
    }
} %c% "stab.Rd"
```

Now we will assign the cell line to each output and plot the counts for the individual stability types.

```
library(ggplot2)

cell_line <- rep(c("HaCaT", "HeLa"), each = 3)
stab$cell_line <- factor(cell_line[stab$basis_idx], levels = c("HaCaT", "HeLa"))
ggplot(stab, aes(x = what, fill = cell_line, group = basis_idx)) +
    stat_count(position = "dodge", col = "black") +
    scale_fill_manual(values = c("royalblue", "red3")) + theme_bw()
```

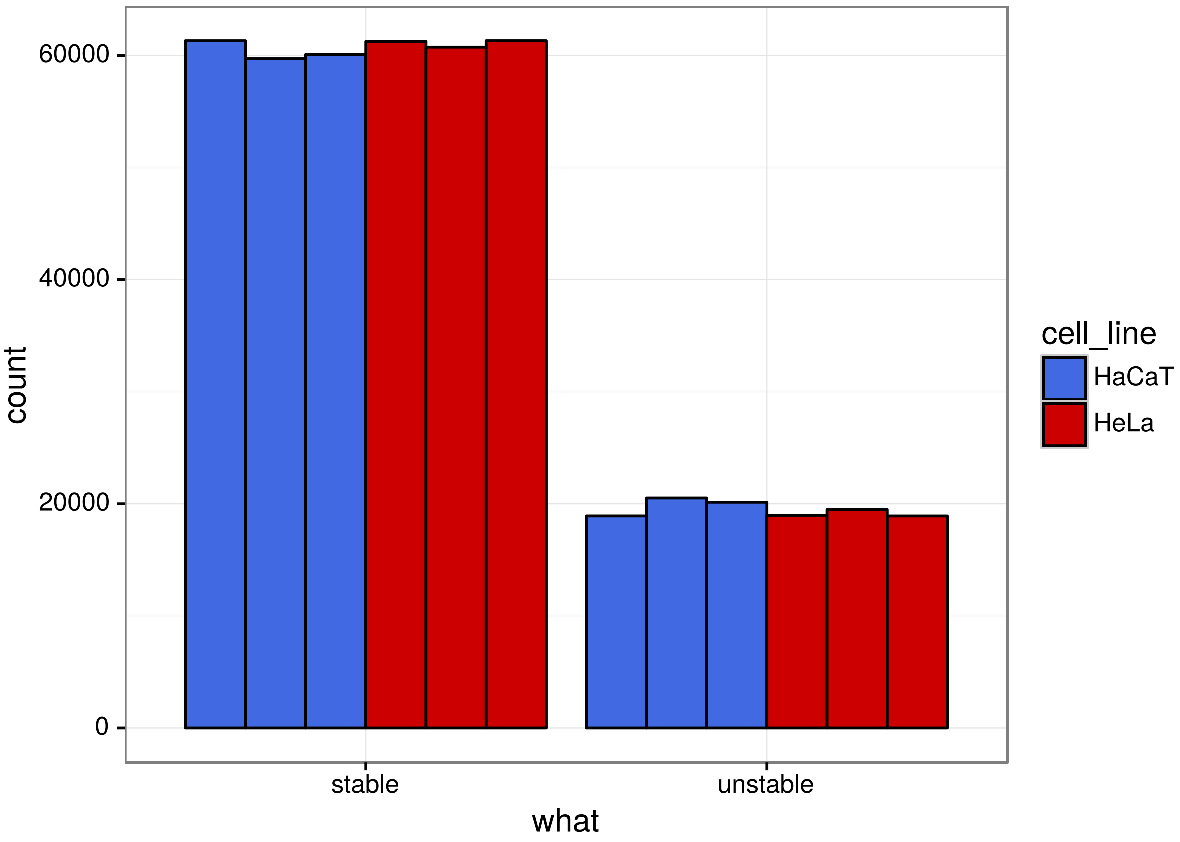

Differential activities

The statistical tests used in `dycone` assume a log-normal distribution of the metabolic terms. It is sufficient to show an approximate normal distribution for the log-transformed metabolite measurements since the log-transform of the metabolic terms is a weighted sum of the logarithmic metabolite measurements for most of the common kinetics (such as mass-action, Michalis-Menten and Hill). We will compare distribution to a normal one via quantile-quantile plots and the empirical distribution of the data.

```
log_all <- log(metab[, 5:10], 2)
colnames(log_all) <- c(paste0("HaCaT_", 1:3), paste0("HeLa_", 1:3))
log_hacat <- unlist(log_all[, 1:3])
log_hela <- unlist(unlist(log_all[, 4:6]))

par(mfrow = 1:2)
qqnorm(log_hacat, pch = 1, col = "royalblue", main = "")
qqline(log_hacat, lwd = 2, col = "darkblue")
points(qqnorm(log_hela, plot.it = F), pch = 2, col = "red3")
qqline(log_hela, lwd = 2, col = "darkred")

x <- seq(0, 16, length.out = 256)
plot(ecdf(log_hacat), pch = 1, col = "royalblue", main = "")
lines(x, pnorm(x, mean(log_hacat, na.rm = T), sd(log_hacat, na.rm = T)), lwd = 2,
    col = "darkblue")
plot(ecdf(log_hela), pch = 2, add = T, col = "red3")
lines(x, pnorm(x, mean(log_hela, na.rm = T), sd(log_hela, na.rm = T)), lwd = 2,
    col = "darkred")
```

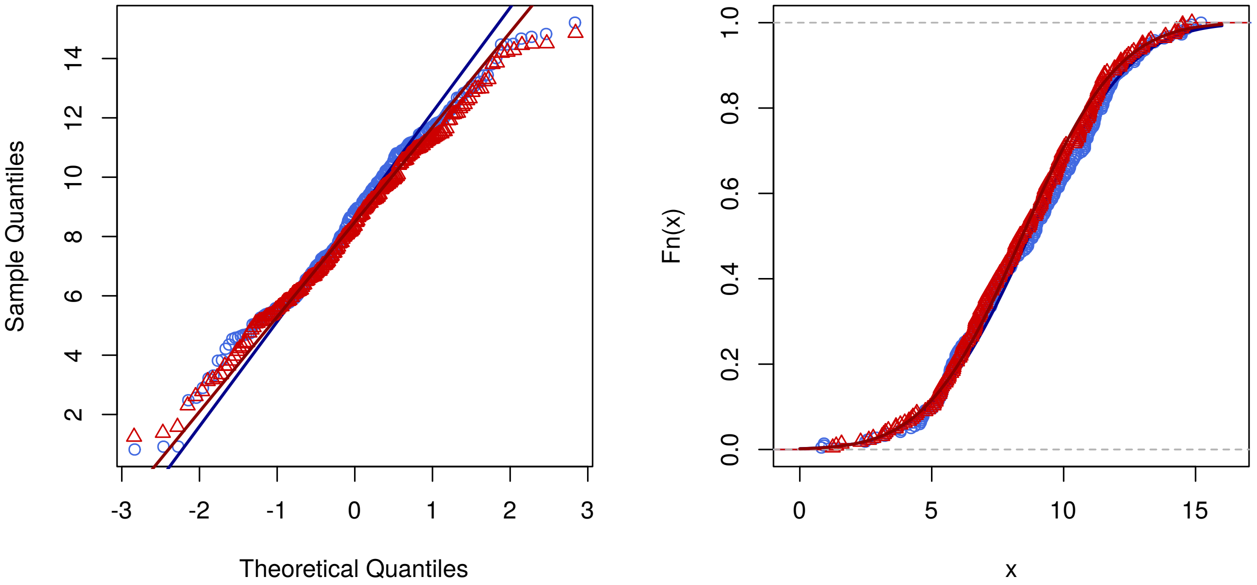

The data is approximately normal in log-space so we can continue with the analysis.

We already calculated the log-fold changes before, but in order to also assign some statistics to that we can also use the function `hyp` from `dycone` which does that for us. It will generate all log-fold changes between disease and normal measurements and perform an empirical Bayes version of t-test. The output will be sorted by increasing p-values and mean log-fold changes. We will also use the `full` option to obtain the raw log-fold changes and append some additional annotations to the result using the `rp` function.

```
h <- hyp(mats[, 1:3], mats[, 4:6], r, full = T)
```

```
## Warning: Zero sample variances detected, have been offset
```

```
pw <- rp(make_irreversible(r), "pathway")[,2]
r_ids <- rp(make_irreversible(r), "KEGG_reaction")[,2]
h$hyp <- cbind(h$hyp, pathway = pw[h$hyp$idx])
h$hyp <- cbind(h$hyp, reaction_id = r_ids[h$hyp$idx])
write.csv(h$hyp, file = "transform.csv", quote = F, row.names = F)
```

`h$hyp` now contains the expected differential activities for each reaction.

```
head(h$hyp)
```

```
##    idx  name                              reaction type  sd_normal
## 11  11   PFK         1*atp + 1*f6p -> 1*adp + 1*fdp   up 0.04180208
## 13  13   GPI                        1*f6p -> 1*g6p   up 0.17043926
## 39  39 TALDO         1*f6p + 1*e4p -> 1*g3p + 1*s7p   up 0.17043926
## 43  43   TKT      1*f6p + 1*g3p -> 1*xu5p-D + 1*e4p   up 0.17043926
## 51  51 ICDH1     1*icit + 1*nadp -> 1*akg + 1*nadph down 0.30874187
## 67  67    PC 1*pyr + 1*atp -> 1*oaa + 1*adp + 1*pi   up 0.14576925
##    sd_disease mean_log_fold   ci_low   ci_high        pval  corr_pval
## 11  0.6374241      3.004645 2.666972  3.433076 0.001269880 0.04228564
## 13  0.5184869      1.690130 1.404087  1.981428 0.005497133 0.04228564
## 39  0.5184869      1.690130 1.404918  2.006415 0.005497133 0.04228564
## 43  0.5184869      1.690130 1.429117  2.006192 0.005497133 0.04228564
```

```
## 51  0.3654610     -1.408384 -1.636298 -1.166145 0.002860691 0.04228564
## 67  0.3539851      1.314515  1.087074  1.503810 0.003312277 0.04228564
##               pathway reaction_id
## 11        Glycolysis      R00756
## 13        Glycolysis      R00771
## 39 Pentose phosphate      R08575
## 43 Pentose phosphate      R01067
## 51         TCA cycle      R00709
## 67         TCA cycle      R00214
```

Those values are the basis for Figures 3 and 4.

Worst-case analysis via linear programming

The analysis we will perform here is very similar to that in the previous section, however, this time we will obtain the differential enzyme activities by correcting for effects that can be caused by flux variation. For this we will analyze the smallest and largest flux for each reaction that still allows a given biomass/growth flux to operate at its optimum. Using `dycone` the only explicit action required of the user is definition of the optimization criterion. For this we will first define all metabolites which are precursors for compounds required for proliferation. Each of those will be assigned a weight which is obtained from the stoichiometry of Recon 2 biomass reaction (http://www.ebi.ac.uk/biomodels-main/MODEL1109130000). For the cases where one metabolite is the precursor for several proliferation compounds we use the maximum stoichiometry from Recon 2. Negative stoichiometries denote compounds that are consumed and positive stoichiometries denote produced compounds. Here we only produce ADP and Pi to balance the ATP usage. The small difference in the stoichiometry (20.7045 vs 20.6508) in Recon 2 accounts for the ATP used during DNA replication.

```
prolif <- c(atp = -20.7045, prpp = -0.053446, pyr = -0.50563, oaa = -0.35261,
    glu = -0.38587, cit = -0.15446, `3pg` = -0.39253, adp = 20.6508,
    pi = 20.6508)
```

As we can see there is a large requirement for ATP and amino acid precursors. Also the form here is not the only way to formulate an objective reaction. Please refer to the documentation of `fba` for alternative input forms.

We can now use the `hyp` function again to generate hypotheses for differentially regulated enzymes correcting for fold changes that can be explained by flux variation. This time setting the type to "fva" which will require defining the additional `v_opt` which defines the objective reaction. The output will be same as before only with an additional column "fva_log_fold" denoting the largest absolute fold-change that can be explained by flux variability alone. We will also append additional annotations again and save the output to a CSV file. This will solve a series of linear programming problems (in our case more than 200). To accelaerate this a bit, `hyp` will automatically execute those in parallel if you registered any of the backends compatible with `foreach`. Since we already did that during the stability analysis the following code will automatically run in parallel. Finally, we will save the complete results in `EDAs.csv`.

```
# Generate hypothesis
h <- hyp(mats[, 1:3], mats[, 4:6], r, type = "fva", obj = prolif, full = T)
```

```
## Warning: Zero sample variances detected, have been offset
```

```
pw <- rp(make_irreversible(r), "pathway")[,2]
r_ids <- rp(make_irreversible(r), "KEGG_reaction")[,2]
h$hyp <- cbind(h$hyp, pathway = pw[h$hyp$idx])
h$hyp <- cbind(h$hyp, reaction_id = r_ids[h$hyp$idx])
write.csv(h$hyp, file = "EDAs.csv", quote = F, row.names = F)
```

Let us use visualize those results to also compare the obtained regulations on a pathway level and mark reactions whose expected differential activity log-fold changes can not be explained by flux variability.

```
ggplot(h$hyp, aes(y = pathway, x = mean_log_fold, col = pathway)) +
    geom_vline(xintercept = 0, linetype = "dashed") +
    geom_point(aes(shape = abs(mean_log_fold) > fva_log_fold),
    position = position_jitter(height = 0.2), size = 3) + scale_shape_manual(values = c(1, 17)) +
    theme_bw() + theme(legend.position = "none") + xlab("mean log-fold change") + ylab("")
```

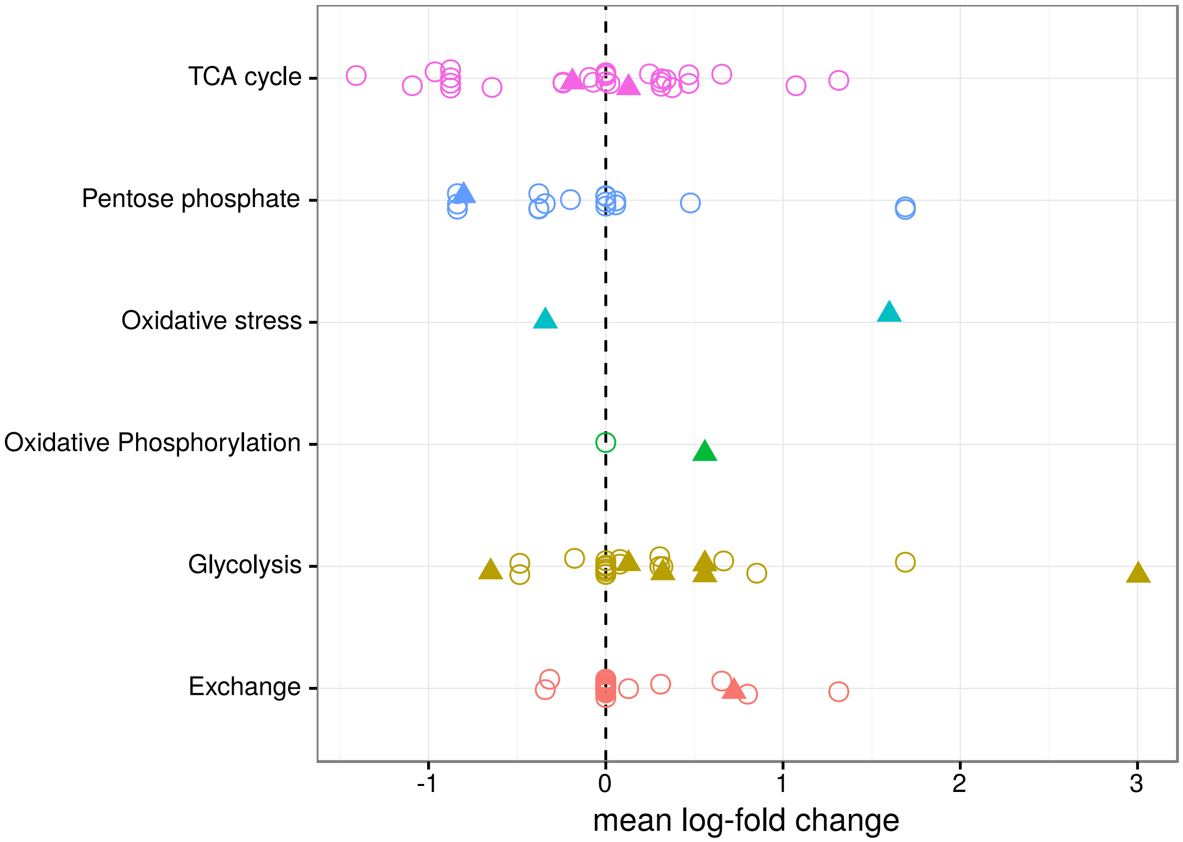

The full output of hyp now contains some additional information. One interesting one are the upper bounds for log-fold changes in the fluxes due to variation.

```
print(h$lfc_va)
```

```
##   [1]  5.315085e+01  5.315085e+01  1.291667e+01  1.014404e-01  4.927036e+01
##   [6]  1.288475e+00  5.239114e+01  0.000000e+00  7.998956e-13  1.242982e+01
##  [11] -6.414869e-13  4.413716e+00  5.293067e+01  1.159063e+01  1.306548e+01
##  [16]  7.939693e-13  4.927036e+01  1.014404e-01  7.372992e-01  5.152572e+01
##  [21]  5.352926e-13  1.288475e+00  5.224029e+01  1.480733e+01  4.285063e-12
##  [26]  7.256418e-13  8.547966e-01  5.198938e+01  6.643242e-12  5.315085e+01
##  [31]  5.057074e+01  6.757134e+00  5.312862e+01  1.464084e+00  5.250142e+01
##  [36]  5.284599e+01  2.392306e+00  2.464084e+00  5.271153e+01  2.464084e+00
##  [41]  5.271153e+01  2.464084e+00  5.267799e+01  2.725973e+00  5.291444e+01
##  [46]  1.432499e-11  8.057042e-01  5.192625e+01  5.315085e+01  5.315085e+01
##  [51]  5.315085e+01  5.315085e+01  1.704836e+00  5.262228e+01  8.057042e-01
##  [56]  5.192625e+01  1.215065e+01  5.364138e-13  1.224596e+00  5.094637e+01
```

```
##  [61]  5.315085e+01  5.315085e+01  5.315085e+01  5.315085e+01  3.880485e+00
##  [66]  5.151465e+01  1.162704e+01  1.035989e+00  5.218596e+01  5.315085e+01
##  [71]  5.306277e+01  5.315085e+01  5.146359e+01  4.118005e-13  6.409736e-12
##  [76]  6.411978e-12  6.397563e-12  3.350653e-13  1.035989e+00  5.218596e+01
##  [81]  6.395000e-12  5.863020e+00  1.550067e+01  6.375299e-12  2.282192e-10
##  [86]  5.313745e+01  6.757134e+00  1.530418e+01  1.317673e+01  1.355539e+01
##  [91]  5.738939e-13  5.216287e+01  1.012125e+00  1.012125e+00  5.216287e+01
##  [96]  5.315085e+01  5.315085e+01  2.573089e+00  4.271449e-12  1.310771e+01
```

We can use those estimates to annotate each of the log-fold changes from the EDAs by the maximum log-fold change estimate from the FVA. Thus, marking reactions whose change in activity is essential for growth.

```
library(pheatmap)
x <- h$lfc_disease
m <- max(range(x))
ann <- data.frame("flux variability" = h$lfc_va, check.names = FALSE)
pheatmap(t(x[h$hyp$idx, ]), breaks = seq(-m, m, length.out = 102), col = dycone::DC_DIVCOL(101),
    cluster_rows = F, cluster_cols = F, cellwidth = 10, cellheight = 10, labels_col = h$hyp$name,
    annotation_col = ann, show_rownames = FALSE)
```

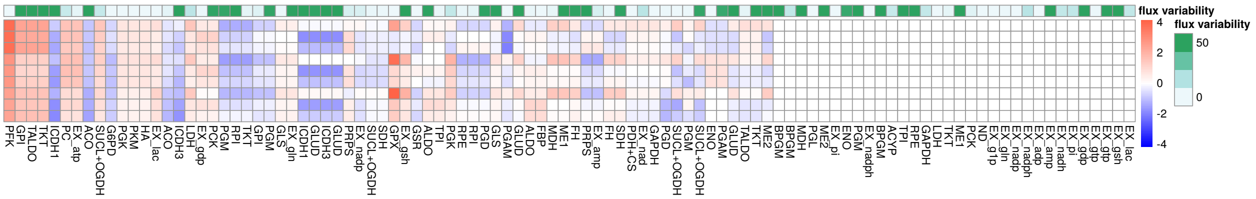

We can also identify significantly altered reactions which are essential directly.

```
essential <- abs(h$hyp$mean_log_fold) > h$hyp$fva_log_fold
h$hyp[h$hyp$corr_pval < 0.05 & essential, ]
```

```
##    idx   name                          reaction type  sd_normal
## 11  11    PFK     1*atp + 1*f6p -> 1*adp + 1*fdp   up 0.04180208
## 26  26   G6PD 1*g6p + 1*nadp -> 1*6pgl + 1*nadph down 0.12433940
## 16  16    PGK   1*13dpg + 1*adp -> 1*3pg + 1*atp   up 0.03160489
## 21  21    PKM     1*adp + 1*pep -> 1*atp + 1*pyr   up 0.03160489
## 74  74     HA              1*pi + 1*adp -> 1*atp   up 0.03160489
## 99  99 EX_lac                      1*lac-L ->    up 0.20775175
##    sd_disease mean_log_fold     ci_low    ci_high        pval
## 11  0.6374241     3.0046450  2.6656965  3.3295961 0.001269880
## 26  0.1903323    -0.8019328 -0.9105587 -0.6874364 0.002003223
## 16  0.1650312     0.5589038  0.4586676  0.6391485 0.004747871
## 21  0.1650312     0.5589038  0.4570282  0.6434021 0.004747871
## 74  0.1650312     0.5589038  0.4551886  0.6448890 0.004747871
## 99  0.2339761     0.7234938  0.6096104  0.8966243 0.006721754
##     fva_log_fold  corr_pval                  pathway reaction_id
## 11 -6.414869e-13 0.04228564               Glycolysis      R00756
## 26  7.256418e-13 0.04228564        Pentose phosphate      R00835
## 16  7.939693e-13 0.04228564               Glycolysis      R01512
## 21  5.352926e-13 0.04228564               Glycolysis      R00200
## 74  4.118005e-13 0.04228564 Oxidative Phosphorylation      R00256
## 99  4.271449e-12 0.04801253                 Exchange        <NA>
```

Reactions that appear strongly regulated in this list can be interpreted as necessary for the given proliferation objective, since there is no flux distribution yielding optimal proliferation without those regulation events.

Heterogeneity and co-regulation

Cancer is known to be a very heterogeneous disease. thus, we might ask what the variation in enzyme activities is within HaCaT and HeLa cells. The hyp output already calculates the standard deviations for the log-fold changes within HaCaT cells and between HeLa and HaCaT cells. So we can easily visualize those.

```
ggplot(h$hyp, aes(x = sd_normal, y = sd_disease, col = pathway)) +
    geom_polygon(data = data.frame(x = c(0, 9, 9), y = c(0, 3, 27)),
    aes(x = x, y = y), fill = "blue", alpha = 0.1, col = NA) +
    geom_abline(color="blue", alpha=0.75) + geom_point() +
    theme_bw() + coord_cartesian(xlim = c(-0.1, 2), ylim = c(-0.1, 2)) +
    xlab(expression(HaCaT ~ sigma)) + ylab(expression(HeLa ~ sigma))
```

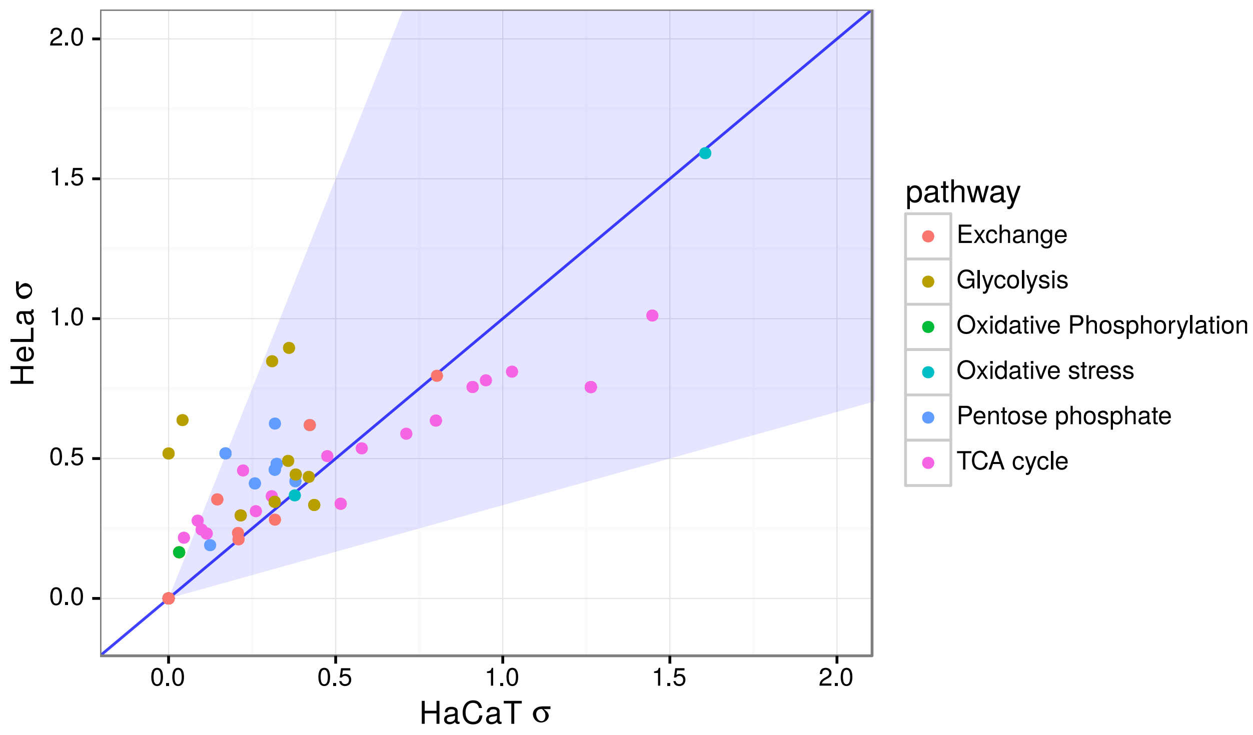

The shaded area correspond to a 3-fold change in both directions (lower and higher) standard deviations. As we can see in the optimized estimates this holds for most pathways, and only a few reactions show higher heterogeneity in cancer (HeLa cells). Let us visualize the correlation between those reactions to see whether they might be co-regulated.

```
fc_sd <- h$hyp$sd_disease/h$hyp$sd_normal

# We ignore fold changes with incomplete data (zero sd in one sample)
sig <- fc_sd > 3 & is.finite(fc_sd)
type <- paste0(h$hyp$name[sig], " (", h$hyp$type[sig], ")")
d <- cor(t(h$lfc_disease[h$hyp$idx[sig], ]))
pheatmap(d, col = dycone::DC_DIVCOL(101), breaks = seq(-1, 1, length.out = 102),
    labels_row = type, labels_col = type)
```

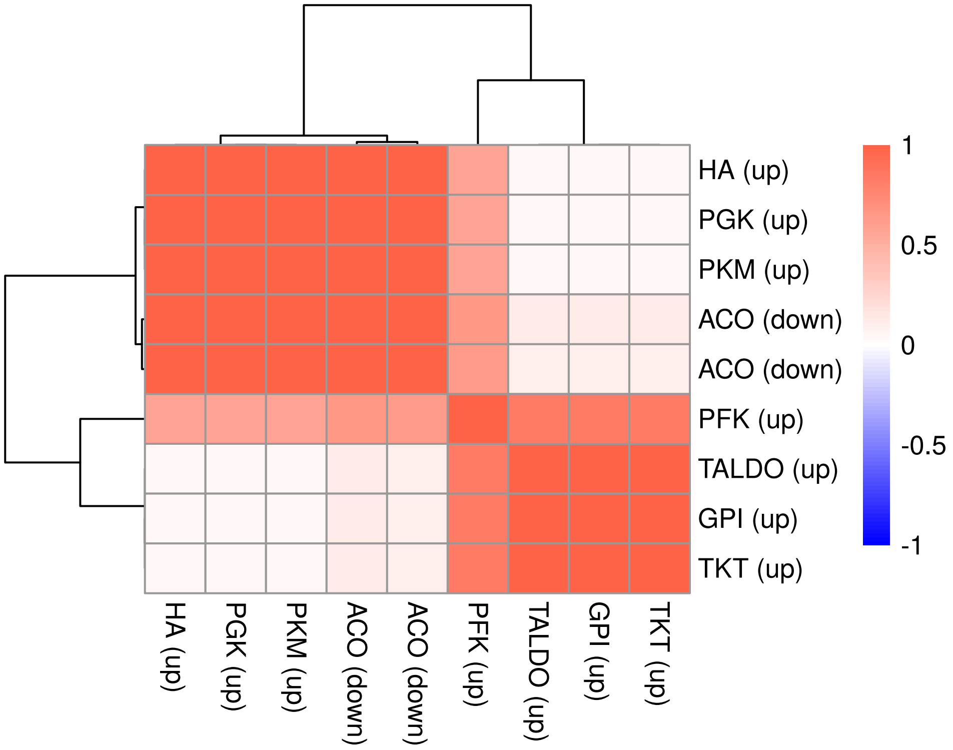

Comparison with gene expression data

Gene expression analysis

We will start by reading a list containing IDs and cell lines for 58 untreated samples from the GEO database and creating an output directory for the downloaded data.

```
sample_info <- read.csv("ge_samples.csv")
dir.create("ma")
```

Since we might run this analysis several times we will first check whether the data is already present and than download all missing data.

```
library(affy, quietly=T, warn.conflicts=F)
library(GEOquery, quietly=T, warn.conflicts=F)

already_there <- dir.exists(paste0("ma/", as.character(sample_info$geoID)))
file_info <- lapply(sample_info$geoID[!already_there], getGEOSuppFiles, baseDir="ma")
```

We will now look for all downloaded raw `.cel` files and also define "normal" and "disease" groups, where HaCaT and keratinocyte arrays will be treated as normal and the HeLa assay as disease.

```
celfiles <- list.files("ma", pattern="*.cel*", recursive=T, ignore.case=T)
names(celfiles) <- sapply(celfiles, dirname)
condition <- rep.int("disease", length(celfiles))
condition[sample_info$cell_line %in% c("HaCaT", "keratinocyte")] <- "normal"
condition <- factor(condition)

table(condition)
```

```
## condition
## disease  normal
##      38      20
```

We will now read the compressed raw data files and assign the phenotype annotations (cell lines and condition).

```
raw_data <- ReadAffy(filenames = paste0("ma/", celfiles[sample_info$geoID]),
    compress=T)
pData(raw_data)$cell_line <- sample_info$cell_line
pData(raw_data)$condition <- condition
```

In order to make the data comparable across arrays we will normalize all of the 58 samples using frozen set RMA.

```
library(frma, quietly=T, warn.conflicts=F)

eset <- frma(raw_data)
```

We will follow this by a short quality control. For this we will check whether the normal and disease samples form condition-specific groups in their expression patterns. As a first try we will visualize the the gene expression patterns in the first two principal components.

```
pca <- prcomp(t(exprs(eset)))
sum(pca$sdev[1:2])/sum(pca$sdev)
```

```
## [1] 0.1760303
```

```
ggplot(data.frame(pca$x), aes(x=PC1, y=PC2)) + theme_bw() +
    geom_point(aes(col=condition, shape=sample_info$cell_line))
```

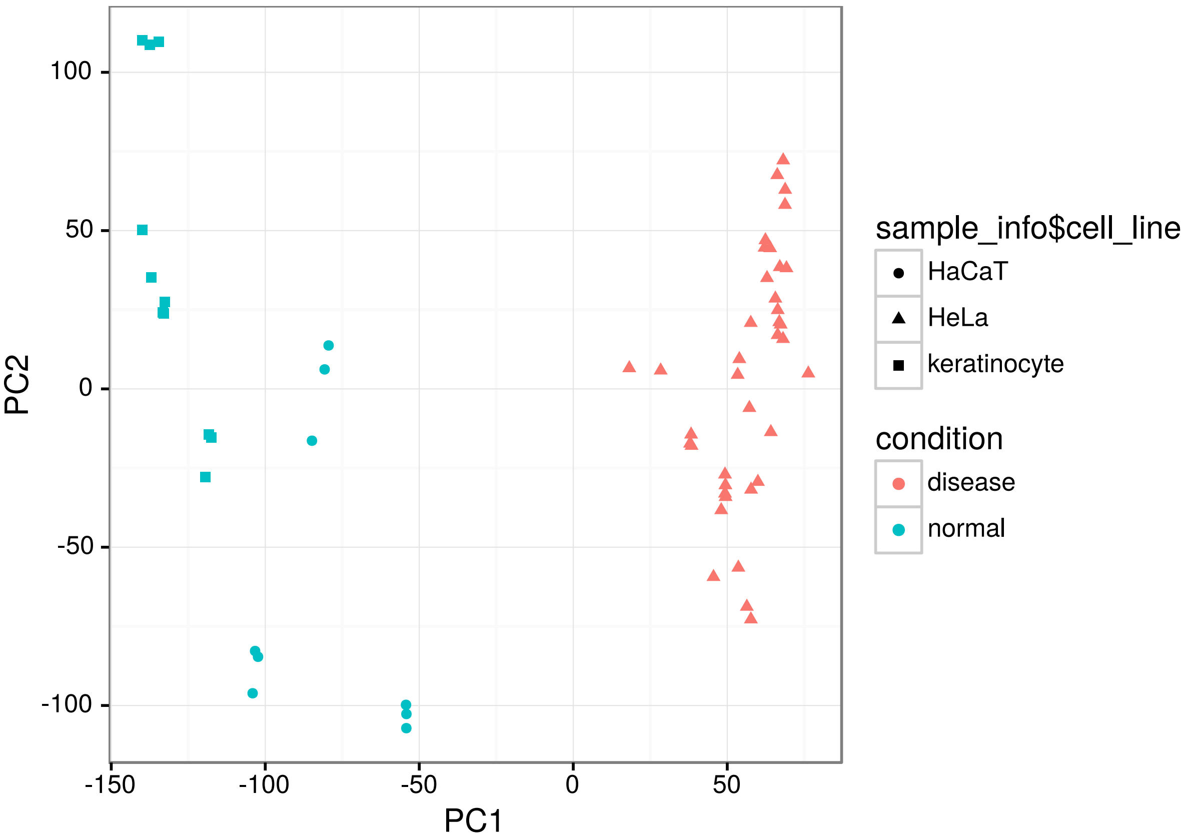

We can also quantify this by first clustering the 58 samples by their expression patterns into two clusters and check how well those two clusters correspond to the condition.

```
cl <- kmeans(t(exprs(eset)), 2)
normal_cluster <- cl$cluster[1] # first condition is normal
cl_err <- sum(cl$cluster[condition == "normal"] != normal_cluster)/length(cl$cluster)
cat(sprintf("Clustering error between normal/disease: %f%%\n", cl_err*100))
```

```
## Clustering error between normal/disease: 0.000000%
```

We will now try to find differentially expressed genes between the normal and disease condition. Since many probes on the array match to the same gene we will choose the probe for each gene which has the maximum mean expression across all 58 samples.

```
library(genefilter, quietly=T, warn.conflicts=F)

mean_max <- findLargest(rownames(eset), rowMeans(exprs(eset)))
gset <- eset[mean_max, ]
rownames(gset) <- as.character(hgu133plus2ENTREZID)[mean_max]
cat(sprintf("Identified genes: %d\n", nrow(gset)))
```

```
## Identified genes: 20546
```

Differential expression will be judged by a t-test where the sample variances are estimated using the empirical Bayes method from the `limma` package. Finally, we will save the gene-wise log2-fold changes along with FDR-corrected p-values to an intermediate data file.

```r
library(limma, quietly=T, warn.conflicts=F)

design <- model.matrix(~ 0 + condition)
colnames(design) <- levels(condition)
fit <- lmFit(gset, design)
contrast.matrix <- makeContrasts(disease - normal, levels=design)
cfit <- contrasts.fit(fit, contrast.matrix)
ebfit <- eBayes(cfit)

ma_lfcs <- topTable(ebfit, number=Inf)
save(ma_lfcs, file="gene_expression.Rd")
head(ma_lfcs)
```

```
##            logFC  AveExpr         t      P.Value    adj.P.Val         B
## 26298  -7.285472 6.106766 -83.65951 1.864868e-62 3.831558e-58 127.81730
## 53836  -5.894125 5.800357 -73.40869 3.560666e-59 3.657873e-55 121.56471
## 999    -7.838556 6.646372 -62.96517 2.463908e-55 1.687448e-51 113.89817
## 153572 -4.679696 5.949331 -61.28846 1.162532e-54 5.971347e-51 112.51945
## 646    -5.391413 4.966523 -50.91969 4.740809e-50 1.948093e-46 102.86099
## 6665   -3.550500 6.694442 -47.15074 3.789863e-48 1.297775e-44  98.77662
```

Comparison to expected differential activity

We will start by mapping all the EC numbers of the reactions used in the model to its respective ENTREZ gene ids and names and saving that information into the `info` data frame. Note that a single reaction might be associated to several EC numbers (isoenzymes for example) and every EC number might be associated with several genes.

```r
library(AnnotationDbi, quietly=TRUE, warn.conflicts=F)
library(dplyr, quietly=TRUE, warn.conflicts=F)

ecs <- rp(make_irreversible(r), "KEGG_enzyme")
ens <- AnnotationDbi::select(hgu133plus2.db, keys = ecs[, 2], keytype = "ENZYME",
    columns = c("SYMBOL", "ENSEMBL", "ENTREZID"))
```

```
## 'select()' returned many:many mapping between keys and columns
```

```r
load("gene_expression.Rd")
info <- ecs %>% group_by(r_idx) %>% do(ens[ens$ENZYME %in% .$KEGG_enzyme,])
```

We will only use those EC numbers for which we could find a corresponding gene on the arrays.

```r
good <- sapply(info$ENTREZID, function(eid) !is.na(eid) &
    (eid %in% rownames(ma_lfcs)))
cat(sprintf("%f%% of enzymes found on array.\n", sum(good)/length(good)*100))
```

```
## 86.206897% of enzymes found on array.
```

```r
info <- info[good, ]
```

Now we will start adding the correponding log fold changes and p-values from the EDAs.

```r
info <- info %>% group_by(r_idx) %>% mutate(met_lfc =
    h$hyp$mean_log_fold[h$hyp$idx %in% r_idx], met_pval =
    h$hyp$corr_pval[h$hyp$idx %in% r_idx], pathway =
    h$hyp$pathway[h$hyp$idx %in% r_idx])
```

And, finally, we will add the log fold changes obtained from the microarrays along with their p-values. The fully assembled `info` data frame will be saved as a csv file.

```r
get_ma_lfc <- function(eid) {
    found <- which(eid[1] == rownames(ma_lfcs))

    return(data.frame(ge_lfc = ma_lfcs$logFC[found],
        ge_pval = ma_lfcs$adj.P.Val[found]))
}

ge <- lapply(info$ENTREZID, get_ma_lfc)
info <- cbind(info, do.call(rbind, ge))
write.csv(info, "all_lfcs.csv")
```

First we will take a look how well the two measurements for enzyme activity coincide in their log fold changes. Significance in EDAs and gene expression is indicated by triangles.

```r
info$significant <- info$ge_pval<0.05 & info$met_pval<0.05
ggplot(info, aes(x=ge_lfc, y=met_lfc, color=pathway,shape=significant)) +
    geom_hline(yintercept=0, linetype="dashed") +
    geom_vline(xintercept=0, linetype="dashed") + geom_point() + theme_bw() +
    xlab("gene expression") + scale_color_discrete(drop=FALSE) +
    ylab("metabolome")
```

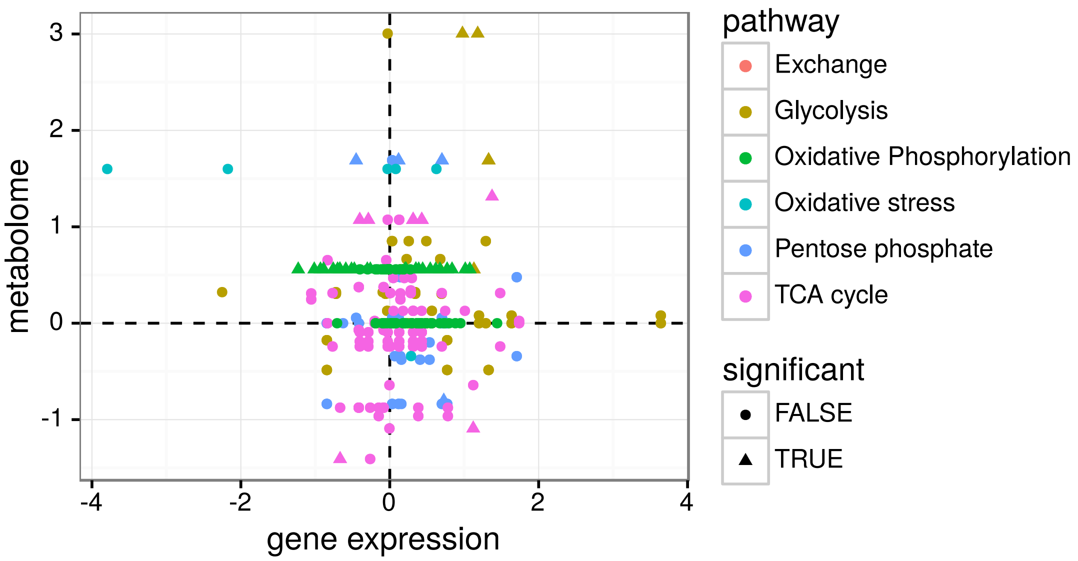

```r
cor.test(info$met_lfc, info$ge_lfc)
```

```
##
##  Pearson's product-moment correlation
##
## data:  info$met_lfc and info$ge_lfc
## t = 0.21481, df = 498, p-value = 0.83
## alternative hypothesis: true correlation is not equal to 0
## 95 percent confidence interval:
##  -0.07813125  0.09723380
## sample estimates:
##         cor
## 0.009625287
```

So there is no correlation on a global level. However, looking at the changes that are significant in EDAs and gene expression we find some cases were gene expression influences the enzymatic activity.

```r
info[info$significant, ]
```

```
##     r_idx   ENZYME   SYMBOL         ENSEMBL ENTREZID   met_lfc   met_pval
## 41     11 2.7.1.11     PFKM ENSG00000152556     5213 3.0046450 0.04228564
## 42     11 2.7.1.11     PFKP ENSG00000067057     5214 3.0046450 0.04228564
## 47     13  5.3.1.9      GPI ENSG00000105220     2821 1.6901302 0.04228564
## 48     13  5.3.1.9      GPI ENSG00000282019     2821 1.6901302 0.04228564
## 49     13  5.3.1.9      GPI ENSG00000105220     2821 1.6901302 0.04228564
## 50     13  5.3.1.9      GPI ENSG00000282019     2821 1.6901302 0.04228564
## 57     16  2.7.2.3     PGK1 ENSG00000102144     5230 0.5589038 0.04228564
## 58     16  2.7.2.3     PGK2 ENSG00000170950     5232 0.5589038 0.04228564
## 59     16  2.7.2.3     PGK1 ENSG00000102144     5230 0.5589038 0.04228564
##                       pathway     ge_lfc      ge_pval significant
## 41                 Glycolysis  1.1811748 1.057940e-12        TRUE
## 42                 Glycolysis  0.9765647 6.629340e-06        TRUE
## 47                 Glycolysis  1.3275529 3.685888e-08        TRUE
## 48                 Glycolysis  1.3275529 3.685888e-08        TRUE
## 49                 Glycolysis  1.3275529 3.685888e-08        TRUE
## 50                 Glycolysis  1.3275529 3.685888e-08        TRUE
## 57                 Glycolysis  0.6777700 4.669467e-07        TRUE
## 58                 Glycolysis  0.2262233 1.020720e-02        TRUE
## 59                 Glycolysis  0.6777700 4.669467e-07        TRUE
##  [ reached getOption("max.print") -- omitted 57 rows ]
```
